# A Dominantly Acting Murine Allele of *Mcm4* Causes Chromosomal Abnormalities and Promotes Tumorigenesis

**DOI:** 10.1371/journal.pgen.1003034

**Published:** 2012-11-01

**Authors:** Bruce N. Bagley, Thomas M. Keane, Vilena I. Maklakova, Jonathon G. Marshall, Rachael A. Lester, Michelle M. Cancel, Alex R. Paulsen, Laura E. Bendzick, Raha A. Been, Scott C. Kogan, Robert T. Cormier, Christina Kendziorski, David J. Adams, Lara S. Collier

**Affiliations:** 1 School of Pharmacy and UW Carbone Cancer Center, University of Wisconsin Madison, Madison, Wisconsin, United States of America; 2 Experimental Cancer Genetics, Wellcome Trust Sanger Institute, Wellcome Trust Genome Campus, Hinxton, United Kingdom; 3 Department of Genetics, Cell Biology, and Development, Masonic Cancer Center, University of Minnesota Twin Cities, Minneapolis, Minnesota, United States of America; 4 Department of Laboratory Medicine and Helen Diller Family Comprehensive Cancer Center, University of California San Francisco, San Francisco, California, United States of America; 5 Department of Biomedical Sciences, University of Minnesota Medical School Duluth, Duluth, Minnesota, United States of America; 6 Department of Biostatistics and Medical Informatics and UW Carbone Cancer Center, University of Wisconsin Madison, Madison, Wisconsin, United States of America; Centre for Cancer Biology, SA Pathology, Australia

## Abstract

Here we report the isolation of a murine model for heritable T cell lymphoblastic leukemia/lymphoma (T-ALL) called *Spontaneous dominant leukemia* (*Sdl*). *Sdl* heterozygous mice develop disease with a short latency and high penetrance, while mice homozygous for the mutation die early during embryonic development. *Sdl* mice exhibit an increase in the frequency of micronucleated reticulocytes, and T-ALLs from *Sdl* mice harbor small amplifications and deletions, including activating deletions at the *Notch1* locus. Using exome sequencing it was determined that *Sdl* mice harbor a spontaneously acquired mutation in *Mcm4* (*Mcm4^D573H^*). MCM4 is part of the heterohexameric complex of MCM2–7 that is important for licensing of DNA origins prior to S phase and also serves as the core of the replicative helicase that unwinds DNA at replication forks. Previous studies in murine models have discovered that genetic reductions of MCM complex levels promote tumor formation by causing genomic instability. However, *Sdl* mice possess normal levels of *Mcm*s, and there is no evidence for loss-of-heterozygosity at the *Mcm4* locus in *Sdl* leukemias. Studies in *Saccharomyces cerevisiae* indicate that the *Sdl* mutation produces a biologically inactive helicase. Together, these data support a model in which chromosomal abnormalities in *Sdl* mice result from the ability of MCM4^D573H^ to incorporate into MCM complexes and render them inactive. Our studies indicate that dominantly acting alleles of MCMs can be compatible with viability but have dramatic oncogenic consequences by causing chromosomal abnormalities.

## Author Summary

Our study investigated a spontaneous mouse model for dominantly inherited T-cell leukemia/lymphoma. Using genetic methods, we identified a mutant allele of *Mcm4* (*Mcm4^D573H^*) in this model. Interestingly, this *Mcm4* allele promotes the accumulation of focal chromosomal gains and losses, including aberrations at the *Notch1* locus that drive the formation of T-cell leukemia/lymphoma. Previous studies of hypomorphic *Mcm* alleles have demonstrated that a decrease in MCM levels can cause tumorigenesis. However, total and chromatin bound MCM levels were similar to wild-type in our model, indicating that *Mcm* alleles that do not drastically impact MCM levels can cause genomic aberrations that drive tumor formation.

## Introduction

Mouse models have been invaluable tools for studying human cancer. Many mouse models used for this purpose are reverse genetic, in that they involve genetically modified mice engineered to have lost a specific tumor suppressor gene (tsg) or to over-express a specific proto-oncogene. More rarely, spontaneous or mutagen induced mouse models that result in tumor formation have been used to study tumorigenesis. Given the contribution of mouse models to understanding tumorigenesis, when a spontaneous mouse mutant that developed T-ALL arose in our colony, we pursued studies to both characterize the disease in these mice and to identify the causal mutation. The mutation was spontaneous and the phenotype dominant, so we named the mutant *Spontaneous dominant leukemia (Sdl)*. We have identified a mutation in the *Mcm4* gene as the likely causative genetic lesion in these mice.

MCM4 is part of the MCM2–7 heterohexameric complex that is involved in licensing origins of DNA replication prior to S phase. The MCM complex has ATPase activity and serves as the core of the replicative helicase that unwinds duplex DNA and drives progression of the replication fork [1]. Improper fork progression can lead to stalled forks, the potential for incomplete DNA replication and even fork collapse which may lead to double strand break (DSB) formation [2]. Therefore, the MCM proteins play important roles in maintaining genomic integrity, however their roles in tumorigenesis are just beginning to be elucidated.

Previous studies of murine *Mcm* genes have involved hypomorphic or gene-trap null alleles. Gene-trap alleles are heterozygous viable and homozygous lethal [3], [4]. Mice harboring hypomorphic alleles of *Mcm2* (*Mcm2^IRES-CreERT2^*) [5] or *Mcm4* (*Mcm4^chaos3^*) [3] show reductions in MCM protein levels and develop tumors only in the homozygous state. *Mcm4^chaos3^* was discovered in a screen for mutations that cause increased micronucleus formation in reticulocytes and therefore promote chromosome instability. *Mcm4^chaos3^* results from a Phe345Ile substitution in MCM4, which is a residue that is involved in the interaction of MCM4 with MCM6 in the heterohexameric complex [3]. In *Mcm4^chaos3/chaos3^* mouse embryonic fibroblasts (MEFs), total and chromatin bound levels of MCM4 and other MCM proteins are reduced compared to wild-type [3], [6]. This leads to a loss of backup origins that normally fire during replicative stress which is hypothesized to be the mechanism by which low levels of MCM proteins promote genomic instability [6], [7]. *Mcm4^chaos3/chaos3^* mice develop tumors with long latency. Although the tumor spectrum varies with genetic background, *Mcm4^chaos3/chaos3^* mice have not been reported to develop T-ALL [3], [6]. We have accumulated evidence that the early-onset T-ALL phenotype in *Sdl* mice results from a novel allele of *Mcm4* (*Mcm4^D573H^*) that in the heterozygous state promotes chromosomal abnormalities that cause highly penetrant tumor formation.

## Results

### *Sdl* causes primarily early onset T-ALL

**Figure 1:**
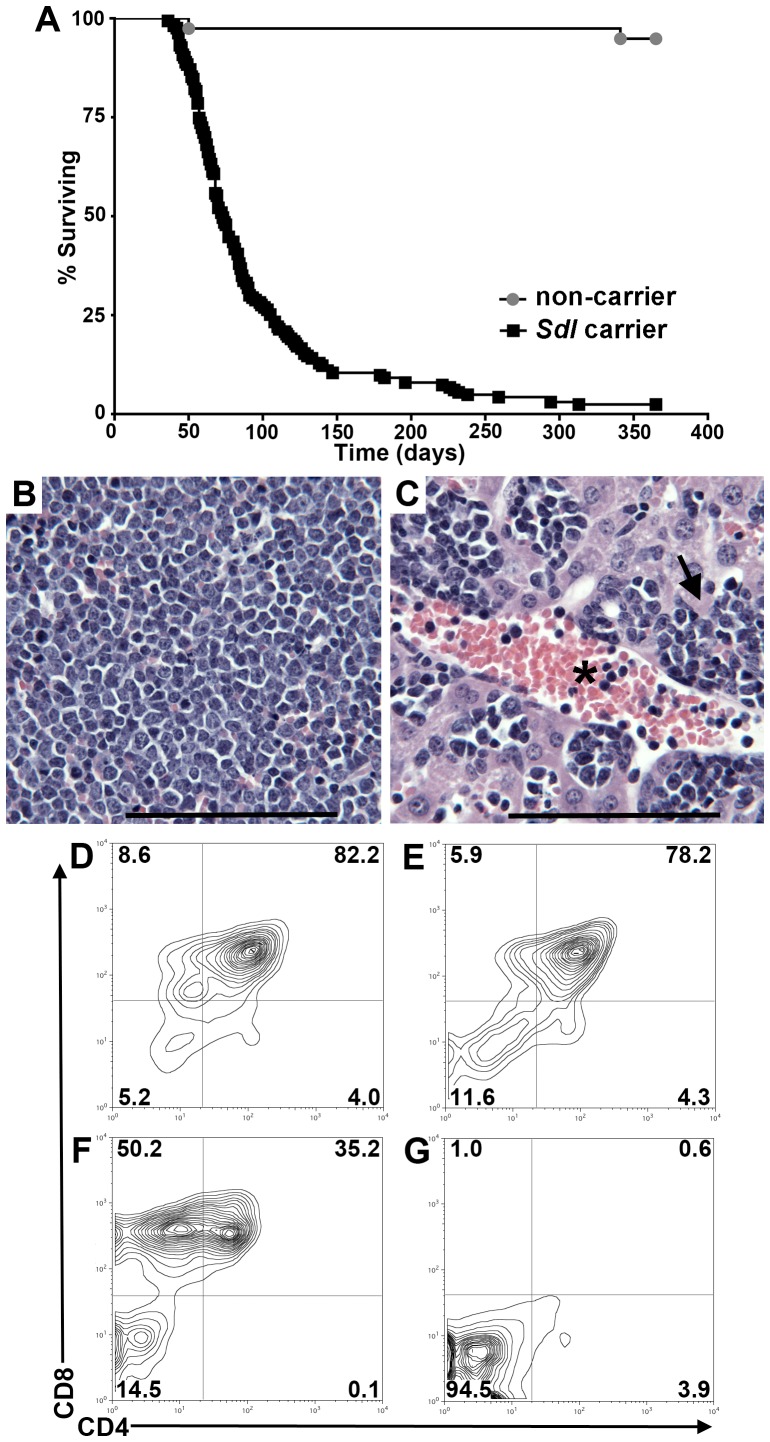
The *Sdl* mutation results in highly penetrant disease, which is primarily early-onset T-ALL. A) Kaplan-Meier curve of time to morbidity for *Sdl* mice. Known carriers of *Sdl* (harboring a C57Bl/6 haplotype at D16MIT131 and D16MIT4 on proximal Chr 16) are denoted by black squares, while sibling non-carriers are denoted by grey circles. p<0.0001. B–C) H&E staining showing that neoplastic cells fill hematopoietic organs (B) and also infiltrate the blood (vessel indicated with an asterisk) and the parenchyma (arrow) of other organs such as liver (C). B and C are 40× magnification, scale bar = 50 µM. D–G) Examples of flow cytometry analysis of lymphomas from four moribund *Sdl* mice. A full summary of flow-cytometry data is available as Table S1. Mice depicted in D–F succumbed to early onset-disease that is phenotypically T-ALL. Within these animals, there is evidence of both CD4/8 double positive (DP) disease as well as CD8 single positive (SP) disease. The mouse in G became moribund with late-onset disease (354 days of age) and the tumor cells do not express most T cell antigens (see also Table S1).

The *Sdl* mutation arose in our colony in the germline of a breeder on the C57Bl/6 genetic background. We therefore pursued a recombination mapping strategy by utilizing out-crosses and backcrosses to the FVB/N and 129S1/SvImJ genetic backgrounds. A whole genome scan using simple sequence length polymorphisms (SSLPs) was performed and it was determined that mice of backcross generations that inherited C57Bl/6 markers at D16MIT131 and D16MIT4 on proximal Chr 16 rapidly became moribund (Figure 1A) indicating linkage to this chromosomal location. Therefore, *Sdl* carriers can be identified by the presence of C57Bl/6 markers at these two SSLPs. Phenotypically, 94.2% (180 of 191) of moribund *Sdl* mice necropsied had signs of hematologic malignancy including mediastinal masses, splenomegaly and/or lymphadenopathy. Histologically, neoplastic cells filled hematopoietic tissues (Figure 1B) and infiltration of neoplastic lymphocytes into non-hematopoietic organs was frequently observed (Figure 1C). Leukemic cells are also found in the blood (Figure 1C) and bone marrow (Figure S1A). *Sdl*-induced disease was transplantable as tumors (Figure S1B) developed with an average latency of 29 days in four of four immunocompromised *nude/nude* mice that received cells isolated from mediastinal masses from moribund *Sdl* mice.

Flow cytometry was used to determine the phenotype of hematologic tumors from four *Sdl* mice. Three mice developed disease early in life that was phenotypically T-ALL (Figure 1D–1F and Table S1). The fourth mouse developed leukemia/lymphoma late in life, which expressed few lineage markers (Figure 1G and Table S1). Southern analysis of early-onset leukemias/lymphomas from *Sdl* mice detects rearrangements of the T cell receptor (TCR) β locus [8] (Figure S1). The majority of *Sdl* leukemias/lymphomas express TdT (Figure S1) that, together with the surface phenotypes, indicates that most *Sdl* mice develop T-ALL with an immature phenotype.

Inter-crosses of *Sdl* heterozygotes were performed to determine the phenotype of *Sdl* homozygotes. No *Sdl/Sdl* mice were present at weaning, so embryos from timed pregnancies of *Sdl* inter-crosses were examined. No *Sdl/Sdl* embryos were detected even as early as 8.5 dpc (n = 69; 20 wild-type, 49 *Sdl*/+, 0 *Sdl/Sdl*; p<0.0001 Chi square test), indicating that *Sdl* is homozygous lethal early during embryonic development. Therefore, all carrier mice utilized for the experiments described here are *Sdl*/+.

### *Sdl* causes subtle defects during thymic development

To further characterize the molecular basis of leukemogenesis in *Sdl* mice, microarray analysis was performed to detect mRNA expression differences between wild-type thymuses and thymuses from pre-leukemic *Sdl* carriers. To detect differentially expressed genes, the false discovery rate was controlled at 5%. Specifically, transcripts with a posterior probability of differential expression >95% and a q-value <0.05 were considered to be significantly differentially expressed (see Methods). No transcripts were found to be significantly differentially expressed between wild-type and pre-leukemic *Sdl* carrier thymuses. Tests for common function did identify Gene Ontology (GO) sets enriched for differential expression between wild-type and carrier thymuses (Table 1), indicating some molecular differences between wild-type and carrier thymuses. However, a similar analysis of the Kyoto Encyclopedia of Genes and Genomes (KEGG) failed to detect any differences between wild-type and carriers.

**Table 1:** Enriched Gene Ontology (GO) Terms between 21-day-old wild-type and carrier thymus.

GO Term	# of genes	Z score
protein localization in mitochondrion	18	12.612
protein targeting to mitochondrion	18	12.612
C-C chemokine binding	17	10.805
C-C chemokine receptor activity	17	10.805
positive regulation of endothelial cell proliferation	16	10.403
mitochondrial transport	39	9.838
chemokine binding	22	9.334
chemokine receptor activity	22	9.334
G-protein chemoattractant receptor activity	22	9.334
regulation of endothelial cell proliferation	20	9.272
endothelial cell proliferation	22	8.863
regulation of immunoglobulin production	29	8.26
positive regulation of angiogenesis	33	7.651
nucleoside diphosphate kinase activity	9	7.358
UTP metabolic process	9	7.358
CTP metabolic process	9	7.358

To determine if *Sdl* impacts T cell development, flow cytometry was performed to characterize T cell developmental stages in *Sdl* carriers. Thymocytes were analyzed from *Sdl* carrier (n = 4) and wild-type siblings at 3.5 weeks of age (n = 4). Analysis of more mature thymocyte populations (CD4, CD8 and CD4/8 double positive) revealed a trend toward decreased levels of CD4+ cells in *Sdl* mice, however this did not reach statistical significance (p<0.064 Table 2). Lineage markers as well as CD44 and CD25 were then utilized to further analyze more immature double negative (DN) populations. There was a statistically significant decrease in the percentage of DN cells at the DN1 stage of development in *Sdl* mice (Table 2). Although no statistically significant differences in other DN cell populations were observed, flow cytometry profiles from individual mice revealed inter-animal differences, particularly in the DN3 population, in *Sdl* mice (Figure S2). Taken together, these data indicate that *Sdl* does cause subtle defects in thymocyte development; with some mice more severely affected than others. However, it is unlikely that *Sdl* causes T-ALL by directly promoting a block in thymocyte differentiation.

**Table 2:** T cell development in wild-type and *Sdl* carrier mice.

	Percent of thymus	
	WT^1^	C
CD4 SP	11.55+/−1.25	8.68+/−1.54^2^
CD4/8 DP	82.85+/−1.71	84.50+/−0.50
CD8 SP	1.91+/−0.34	1.67+/−0.17
DN^3^	1.57+/−0.15	2.72+/−1.40

	Percent of DN	
	WT	C
DN1	12.75+/−2.04	6.11+/−2.61^4^
DN2	4.35+/−0.82	4.04+/−0.78
DN3	40.65+/−2.41	49.75+/−12.67
DN4	42.28+/−1.98	40.08+/−10.52

1 Abbreviations used: WT = wild-type, C = Sdl carrier, SP = single positive, DP = double positive, DN = double negative.

2 t-test p value <0.064.

3 DN cells are defined as negative for all lineage markers (see Materials and Methods).

4 t-test p value <0.014.

### *Sdl* mice harbor a mutation in *Mcm4* that causes chromosomal abnormalities

**Figure 2:**
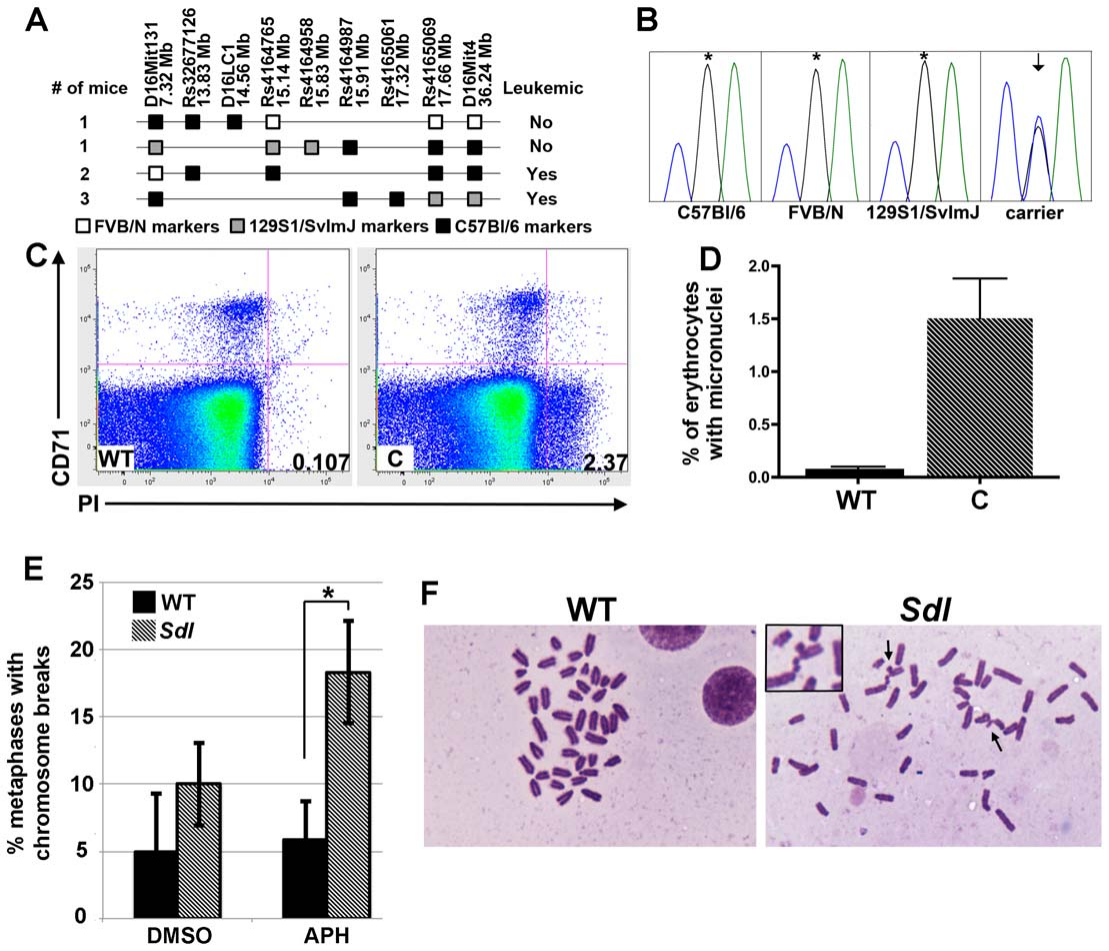
*Sdl* mice harbor a mutation in *Mcm4* that causes chromosomal abnormalities. A) Recombination events in Chr 16 that define the *Sdl* interval. Only the relevant *Sdl* haplotype is shown. White squares: FVB/N alleles, Grey squares: 129S1/SvImJ alleles, Black squares: C57Bl/6 alleles. The *Sdl* mutation must map within regions harboring C57Bl/6 alleles in leukemic mice, but must be excluded from regions harboring C57Bl/6 alleles in non-leukemic mice. Analyzing two non-leukemic mice indicates that the *Sdl* mutation likely lies distal to 14.56 and proximal to 15.91. The two non-leukemic mice were bred and non-carrier status was verified. B) Sanger sequencing traces demonstrating the G to C substitution present in all confirmed *Sdl* carriers but absent from all wild-type strains examined. Arrow indicates dual G/C peak, asterisk indicates wild-type G peak. C) *Sdl* mice harbor increased numbers of spontaneous micronuclei. Example flow cytometry plots for wild-type (WT) and *Sdl* carrier (C) mice for micronuclei detection. Micronucleated normochromatic erythrocytes are propidium iodide (PI) positive but CD71 negative (lower right quadrant) and are expressed as a percentage of total erythrocytes. D) Quantification of micronuclei for sex and aged-matched wild-type (solid bar, WT) (n = 8) and *Sdl* carriers (striped bar, C) (n = 5). Mean for wild-type = 0.0815 and for *Sdl* carriers = 1.503 (p<0.0005). Error bars represent standard deviation. E) *Sdl* (striped bars) and wild-type (solid bars) MEFs were treated with Dimethyl sulfoxide (DMSO) as vehicle control or 0.15 µM aphidicolin (APH) and the percent of metaphases with chromosome breaks determined. No statistically significant difference between *Sdl* and wild-type was observed when cells were treated with vehicle only (p>0.26, t-test). In the presence of APH, *Sdl* MEFs did harbor more chromosome breaks (average 18.3%) than wild-type MEFs (average 5.8%) (p<.02, t-test, asterisk). Error bars represent standard deviation. F) Example metaphase spreads without chromosome breaks (WT, APH treated) and with chromosome breaks (*Sdl*, APH treated). Arrows indicate chromosome breaks, one example shown as inset.

To identify the affected gene in *Sdl* mice, the chromosomal location of the *Sdl* mutation was further narrowed utilizing single nucleotide polymorphic (SNP) markers to analyze mice with recombination events in proximal Chr 16. Using this approach, the *Sdl* mutation was mapped to a 1.4 Mb candidate region (Figure 2A) that contains 30.5 kb of annotated protein-coding sequence. No differences in expression levels of genes in the interval were detected by quantitative RT-PCR (qRT-PCR) analysis comparing 21-day-old *Sdl* carrier thymuses to 21-day-old control thymuses (Figure S3). Exon capture followed by re-sequencing was performed on *Sdl* genomic DNA on the 129S1/SvImJ congenic background. To ensure complete coverage in the *Sdl* interval, PCR amplification followed by Sanger sequencing was used to further examine exon and splice site sequences with fewer than 10× coverage following exon capture (Figure S4 and Table S2). After eliminating known SNPs between C57Bl/6 and 129S1/SvImJ (Table S3), only one non-synonymous sequence difference in the *Sdl* interval was identified. This difference is a G to C missense mutation that causes a D573H substitution in *Mcm4* (*Mcm4^D573H^*). This residue is conserved not only in MCM4 but also across all MCM2–7 subunits in eukaryotes (Figure S5). This nucleotide change was present in all confirmed leukemic *Sdl* mice examined and was not detected in FVB/N, 129S1/SvImJ, or mice of the C57Bl/6 genetic background that were present in the colony at the time that *Sdl* arose (Figure 2B). A cross of *Sdl/+* to *Mcm4^chaos3/+^* did not produce any *Sdl/Mcm4^chaos3^* viable pups at p1 (n = 26; 7 wild-type, 10 *Sdl*/+, 9 *Mcm4^chaos3^* ^/+^, and 0 *Sdl/Mcm4^chaos3^*; Chi square p value 0.0246), indicating non-complementation.

To further investigate if *Sdl* mice harbor phenotypes indicative of replicative stress, chromosomal aberrations were examined in both reticulocytes and mouse embryonic fibroblasts (MEFs) isolated from *Sdl* mice. *Sdl* mice harbor an ∼18-fold increase in spontaneous micronucleated reticulocytes compared to non-carrier siblings (Figure 2C and 2D). This is similar to the ∼20-fold increase reported for *Mcm4^chaos3/chaos3^* mice studied on a different strain background [3]. MEFs from *Sdl* carriers and non-carrier siblings were analyzed cytogenetically for chromosome breaks in the presence and absence of the DNA replication inhibitor aphidicolin (APH) (Figure 2E–2F). More chromosome breaks were found in APH-treated *Sdl* MEFs compared to wild-type (p<0.02). Together, these observations indicate that *Sdl* causes chromosomal aberrations and increased sensitivity to exogenous replication stress, a phenotype that is consistent with MCM dysfunction [3], [5]. Therefore, all evidence suggests that the *Sdl* phenotype is caused by *Mcm4^D573H^*.

### *Mcm4^D573H^* acts in a dominant manner to promote tumorigenesis

**Figure 3:**
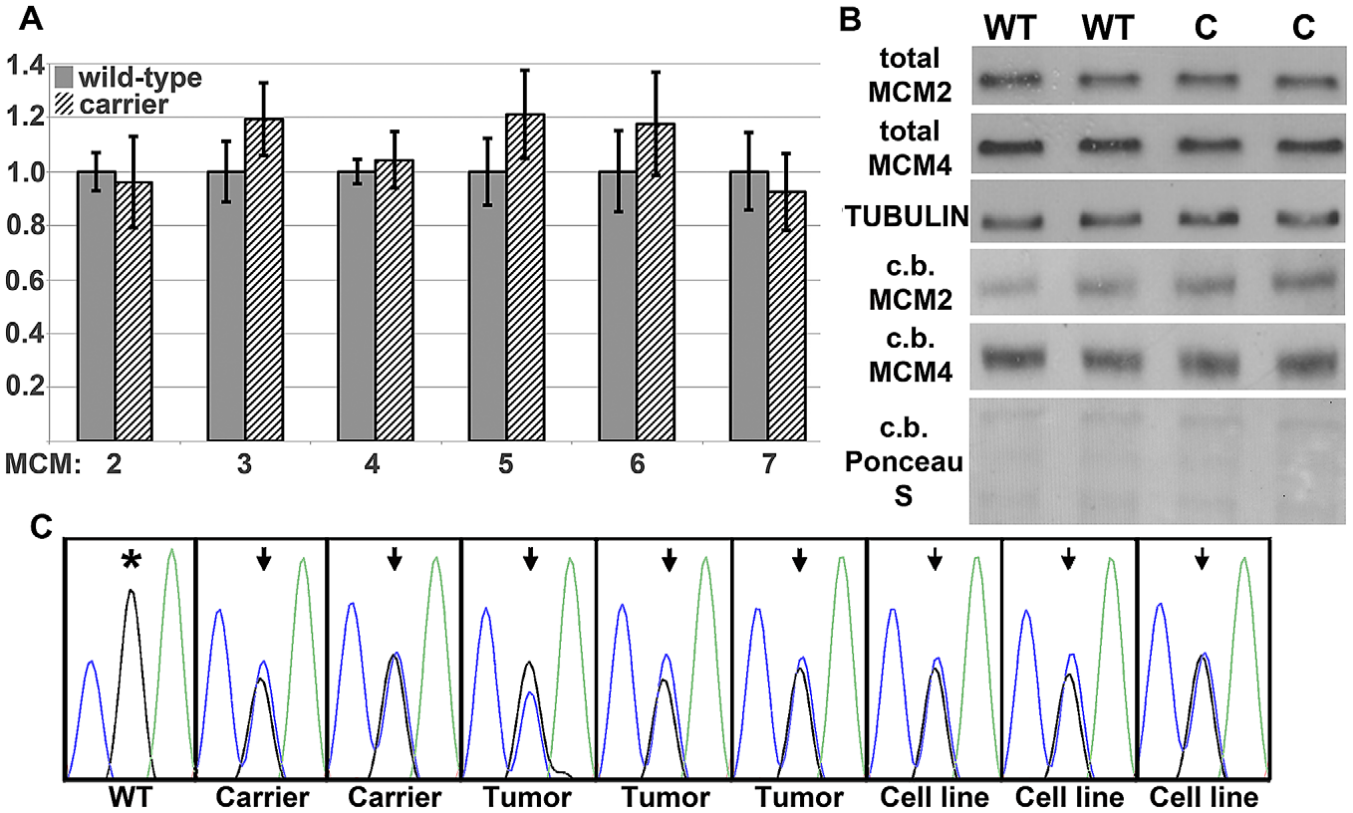
*Mcm4^D573H^* acts in a dominant manner to promote tumorigenesis. WT = wild-type C = carrier. A) *Mcm2–7* transcript levels are not decreased in *Sdl* carrier thymuses (striped bars) compared to wild-type thymuses (solid bars) as analyzed by qRT-PCR. Values for wild-type thymus are normalized to 1. N = 3 for wild-type, 6 for carrier. Error bars represent standard deviation. There is a trend toward increased expression of *Mcm3* and *Mcm5* in *Sdl* carrier thymuses compared to wild-type thymuses (p = 0.07 and 0.09, respectively); all other p values >.2. B) Western analysis on total thymus protein extract as well as purified chromatin bound (c.b.) fractions indicate that *Sdl* carrier thymuses harbor similar levels of MCM2 and 4 proteins as do wild-type thymuses. TUBULIN and Ponceau S membrane staining were utilized to demonstrate equal loading for whole cell lysates and chromatin bound fractions, respectively. C) Sanger sequencing traces of RT-PCR products demonstrate that both wild-type (G) and mutant (C) *Mcm4* alleles are expressed in *Sdl* tumors and tumor-derived cell lines. RT-PCR products from 21-day-old wild-type and *Sdl* carrier thymuses are shown for reference. Arrow indicates dual G/C peak, asterisk indicates wild-type G peak.

Previously studied hypomorphic or gene trap null alleles of *Mcm*s have indicated that minimum thresholds of MCM levels are needed for normal development and for tumor suppression in adults; and reductions in protein levels of other members of the MCM2–7 complex have been detected in *Mcm2* and *Mcm4* hypomorphic mice [3]–[6]. Therefore qRT-PCR and Western analyses were utilized to examine *Mcm* levels in 21-day-old wild-type thymuses and 21-day-old *Sdl* carrier thymuses. No reductions in mRNA levels for *Mcm2–7* (Figure 3A) or total or chromatin bound protein levels for MCM2 and MCM4 (Figure 3B) were detected. MCM4 total and chromatin bound levels were also not reduced in *Sdl* MEFs compared to wild-type MEFs (Figure S6). Therefore *Mcm4^D573H^* does not promote tumorigenesis by simply causing a reduction in transcript or protein levels of *Mcm4* or other *Mcm*s.

Although genetically it acts dominantly, *Mcm4^D573H^* could actually promote tumor formation in a recessive manner if loss-of-heterozygosity (LOH) or epigenetic silencing at the *Mcm4* locus occurs during tumor formation. To address these possibilities, RT-PCR followed by re-sequencing was used to examine if both wild-type and mutant *Mcm4* alleles are expressed at the mRNA level in *Sdl* tumors. Peak heights of Sanger sequencing traces indicated that both alleles are expressed at similar levels in both *Sdl* tumors and in 21-day-old thymuses from *Sdl* carrier mice (Figure 3C). As stromal cells are present in bulk tumors, *Mcm4* allele expression was also examined in cell lines that were established from *Sdl* T-ALLs. Both alleles were expressed at similar levels as they are in thymuses from pre-leukemic *Sdl* carrier mice (Figure 3C). Therefore, tumorigenesis in *Sdl* mice does not require LOH, and the *Mcm4^D573H^* allele acts dominantly to cause T-ALL.

### The *Sdl* mutation generates a biologically non-functional helicase

**Figure 4:**
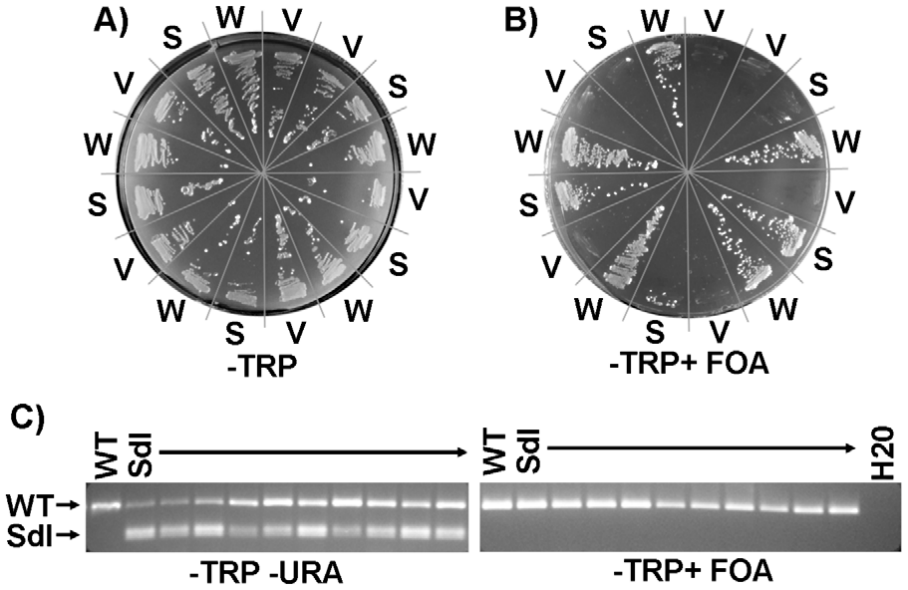
*S. cerevisiae mcm4* engineered with the *Sdl* mutation at the equivalent residue (D632H) generates a non-biologically active helicase. A) Examples of genetic complementation tests of a *mcm4* deletion haploid strain in which viability is maintained by an URA3-*mcm4* plasmid. This strain was transformed with TRP1 plasmids expressing *mcm4^Sdl^* mutation (S), *mcm4* wild-type (W) or empty TRP1 vector (V). A) Growth on permissive conditions (−TRP) demonstrates that all colonies analyzed harbor the expected TRP1 plasmids. B) Growth under restrictive conditions (−TRP+ FOA) occurs only if viability can be maintained by the allele on the TRP1 plasmid. All *mcm4* wild-type colonies grew under restrictive conditions and empty vector colonies do not, as expected. A fraction of colonies expressing *mcm4^Sdl^* mutation grew under restrictive conditions. C) A restriction fragment polymorphism was utilized to distinguish *mcm4^Sdl^* from wild-type (WT) *mcm4* sequences in the yeast strains described above. One *mcm4* wild-type (W) colony and 10 *mcm4^Sdl^* (S) colonies are shown. All freshly isolated *mcm4^Sdl^* colonies grown under permissive conditions (−URA −TRP) harbor both *mcm4^Sdl^* and *mcm4* wild-type sequences due to the presence of TRP1-*mcm4^Sdl^* and URA3-*mcm4* plasmids. All *mcm4^Sdl^* colonies that grew under restrictive conditions lost *mcm4^Sdl^* sequences, indicating that growth occurred due to a reversion or gene conversion event involving *mcm4^Sdl^* sequences on the TRP1 plasmid and not due to the ability of *mcm4^Sdl^* to complement the *mcm4* genomic deletion.

To determine the impact of the *Sdl* mutation on MCM function, complementation studies in *Saccharomyces cerevisiae* were performed. These studies utilized a haploid strain that harbors a deletion of the chromosomal *mcm4* locus in which viability is maintained by a URA3-*mcm4* plasmid [9]. This strain was transformed with a TRP1 plasmid harboring *mcm4* with the *Sdl* mutation engineered into the analogous yeast residue (*mcm4^D632H^*, hereafter referred to as *mcm4^Sdl^*). Cloning into the TRP1 vector added a HA/10XHis tag, which has been shown to not compromise Mcm4 protein function in complementation tests [9] and allowed verification of Mcm4^Sdl^ protein expression by Western blotting (not shown). If the *mcm4^Sdl^* allele expressed by the TRP1 plasmid complements the *mcm4* genomic deficiency, then growth on -TRP+5-Fluoroorotic Acid (FOA) (restrictive conditions) will occur due to the ability to lose the wild-type *mcm4* copy on the URA3 plasmid. TRP1-*mcm4* wild-type and empty TRP1 vectors served as positive and negative controls, respectively. For each TRP1 vector, multiple individual colonies were analyzed for growth under restrictive conditions (example shown in Figure 4A). As expected, no empty TRP1 vector colonies (n = 37) grew while all TRP1-*mcm4* colonies (n = 37) grew. Surprising, *mcm4^Sdl^* showed an intermediate phenotype as 10 of 38 colonies grew. To further examine this phenomenon, *mcm4^Sdl^* colonies were examined for the presence of *mcm4^Sdl^* sequences (Figure 4B). As expected, freshly isolated *mcm4^Sdl^* colonies grown under permissive conditions (−URA −TRP) harbored both wild-type *mcm4* and *mcm4^Sdl^* sequences due to the presence of both URA3-*mcm4* and TRP1-*mcm4^Sdl^* plasmids. However, *mcm4^Sdl^* colonies that grew under restrictive conditions (−TRP +FOA) only harbored *mcm4* wild-type sequences, indicating that a reversion or gene conversion involving *mcm4* sequences on the TRP1-*mcm4^Sdl^* plasmid had occurred. These results indicate that *mcm4^Sdl^* generates a biologically non-functional helicase as it cannot complement a *mcm4* genomic deletion. As *Mcm4^D573H^* does not reduce MCM protein levels in mice, it is hypothesized that it causes chromosomal abnormalities and promotes tumorigenesis by stably incorporating into MCM heterohexamers and interfering with their normal function.

### *Sdl* leukemias are characterized by intragenic deletions at the *Notch1* locus

To further characterize the molecular basis of leukemogenesis in *Sdl* mice, microarray analysis was performed to compare expression in overt thymic tumors from *Sdl* mice to wild-type thymus. Utilizing the same criteria described above for analysis of pre-leukemic *Sdl* carriers, 3627 genes were found to be differentially expressed, of which 745 had ≥2 fold change in expression levels. The 20 significantly differentially expressed genes with the largest fold changes of increased and decreased expression in *Sdl* leukemias compared to wild-type thymuses are outlined in Table 3.

**Table 3:** Top 20 genes with increased and decreased expression in *Sdl* leukemias.

Gene Symbol	q value	PP.LNNMV.DE^1^	fold change
A730037C10Rik	0.022	1	51.653
Susd4	0.032	1	41.487
Cd5l	0.024	1	39.086
Il22	0.045	1	38.299
Fbp1	0.008	1	34.682
Arg1	0.049	1	34.535
Emx2	0.026	1	33.934
Drd5	0.02	1	30.812
Aldh1b1	0.017	1	30.350
Dtx1	0.012	1	28.659
Gm11428	0.043	1	27.660
Drd5	0.019	1	27.146
Ace	0.038	1	23.650
Hmox1	0.009	1	22.894
Wdr25	0.027	1	22.461
Gpnmb	0.034	1	21.205
Heyl	0.015	1	20.901
Adam19	0.036	1	19.015
Hdgfrp3	0.006	1	18.947
Slc16a3	0.005	1	18.561
Ube2l6	0.023	1	0.476
Igfbp5	0.001	1	0.466
Krt8	0	1	0.436
Slc46a2	0.002	1	0.405
Psmb11	0.009	1	0.397
Dgat2	0.008	1	0.378
Gpd1	0.013	1	0.366
Krt18	0	1	0.356
Hpgd	0.001	1	0.310
Akr1c18	0.003	1	0.302
Car3	0.005	1	0.300
Snca	0.035	1	0.258
Cox8b	0.016	1	0.243
Alas2	0.03	1	0.237
Cxcl11	0.004	1	0.192
Skint10	0.008	1	0.170
Ucp1	0.016	1	0.159
Ccl25	0.001	1	0.101
Tbata	0.002	1	0.101
Prss16	0.003	1	0.072

1 posterior probabilities of differential expression (see Materials and Methods for details).

**Figure 5:**
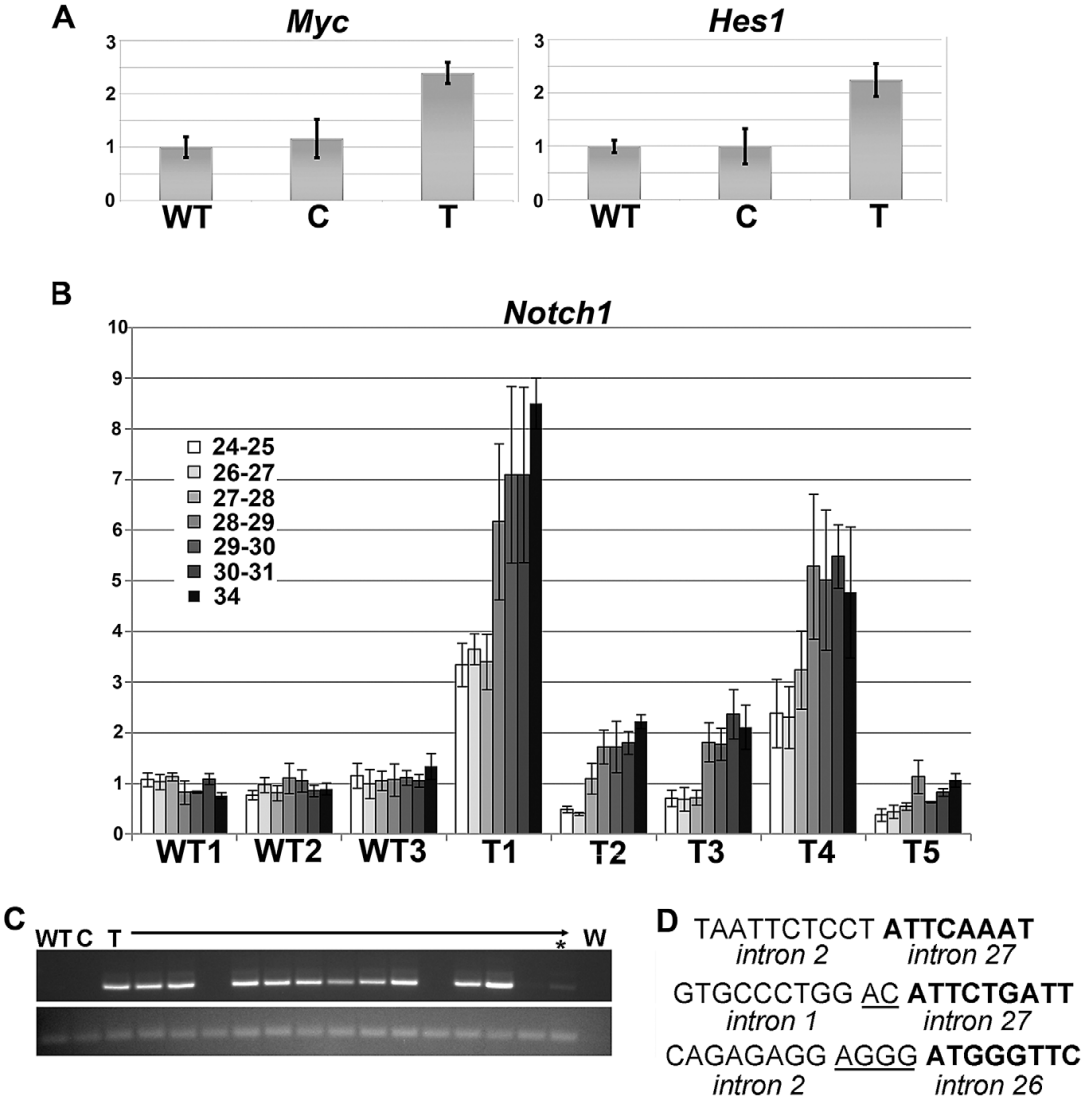
*Notch1* activation due to intragenic deletions occurs during leukemogenesis in *Sdl* mice. WT = wild-type, C = carrier *Sdl* thymus, T = tumor. Error bars represent standard deviation. For all qRT-PCR data, expression in wild-type thymus was normalized to 1. A) qRT-PCR detects higher levels of expression of the *Notch1* targets *Myc* and *Hes1* in *Sdl* thymic tumors compared to wild-type thymus or carrier thymus. N = 3 per group. p<0.001 for comparisons of wild-type thymus or carrier thymus to tumors. B) qRT-PCR results for querying expression levels of individual *Notch1* exon/exon boundaries indicated as well as exon 34 for three wild-type thymuses and five *Sdl* tumors. All q-PCRs were performed in triplicate. C) RT-PCR using a forward primer in exon 1 and a reverse primer in exon 30 detects abnormal *Notch1* transcripts in 11 of 15 tumors (T) from *Sdl* mice but not in wild-type thymus (WT) or thymus from *Sdl* carrier mice (C) (top panel). An additional tumor (asterisk) was weakly positive. A no-RNA control (water, W) is also shown. RT-PCR for *Gapdh* was used to verify the presence of cDNA (bottom panel). D) Sequences surrounding three genomic breakpoints in *Notch1* cloned from *Sdl* tumors. 5′ introns are in regular font, 3′ introns are in bold font. Microhomology is underlined.

Microarray data indicated that the *Notch1* pathway is activated in *Sdl* tumors as *Notch1* itself and several *Notch1* target genes including *Myc, Hes1, Dtx1, Adam19, Hey, Heyl* and *Il2ra* [10]–[13] were transcriptionally up-regulated in *Sdl* tumors compared to normal thymus (Table 3 and microarray data available at GEO). Expression levels of the *Notch1* targets *Hes1* and *Myc* were investigated by qRT-PCR (Figure 5A), which revealed that they were expressed at equivalent levels in wild-type and carrier thymuses, but were approximately 2 fold up-regulated in tumors. As *NOTCH1* activating mutations are present in >50% of human T-ALL [14]; exons 26, 27 and 34 which are the common sites of *Notch1* mutational activation in murine leukemias [15] were sequenced from *Sdl* tumors. Only one point mutation in exon 26 was detected out of 13 tumors sequenced. Therefore, activation of *Notch1* by point mutation is not a common mechanism in this model.

To further investigate the mechanism of *Notch1* activation in *Sdl* T-ALL, *Notch1* transcript levels were investigated by qRT-PCR with primer pairs spanning several exon-exon boundaries (Figure 5B). Five of five *Sdl* tumors examined showed higher levels of expression of 3′ exons than 5′ exons, and in four of five tumors 3′ exons were expressed at higher levels than in normal thymus. These results were consistent with the presence of intragenic deletions removing 5′ regions of the *Notch1* locus that have been recently reported in murine leukemias. These deletions result in truncated or chimeric transcripts that produce NOTCH1 proteins that are constitutively active [16], [17]. Two types of intragenic *Notch1* deletions have been reported in murine T-ALLs. Both types of transcripts were shown to be translated beginning at M1727 in exon 28, produce intracellular NOTCH1 (ICN1) and activate a *Notch1* reporter [16]. Type 1 were more common, had specific break points that occur immediately adjacent to sequences similar to RAG-signal sequences (RSSs) and had features consistent with being driven by RAG activity. Type 2 deletions were more rare (3 of 10 cell lines examined, two of which were sub-clones of the same tumor) and did not have evidence of RSS-like sequences at their breakpoints [16]. Type 1 deletions break at specific chromosomal locations, so genomic PCR can be used to detect them. No such deletions were detected in *Sdl* tumors. Type 2 deletions have varying breakpoints as they are not limited to RSS-like sequences, so they are difficult to detect via genomic PCR. However, RT-PCR can be used to detect the resulting abnormal chimeric transcripts. Such transcripts were detected in 12 of 15 tumors examined (Figure 5C). Sequencing of the primary RT-PCR product from three separate tumors revealed splicing from exon 1 to exon 28. To attempt to clone the breakpoints in *Notch1* in *Sdl* tumors, genomic PCR on a separate cohort of tumors was performed with various forward primers spanning exon 1 through intron 2 in combination with an exon 27 or exon 28 reverse primer. In 3 of 13 tumors, products were cloned and it was verified that the breakpoints do not possess evidence of RSS-like sequences. Two of three breakpoints had 2–4 bp microhomology (Figure 5D). Therefore, it is hypothesized that the tumor spectrum of *Sdl* mice is, at least in part, due to the propensity to develop type 2 non-RAG driven deletions at the *Notch1* locus.

**Figure 6:**
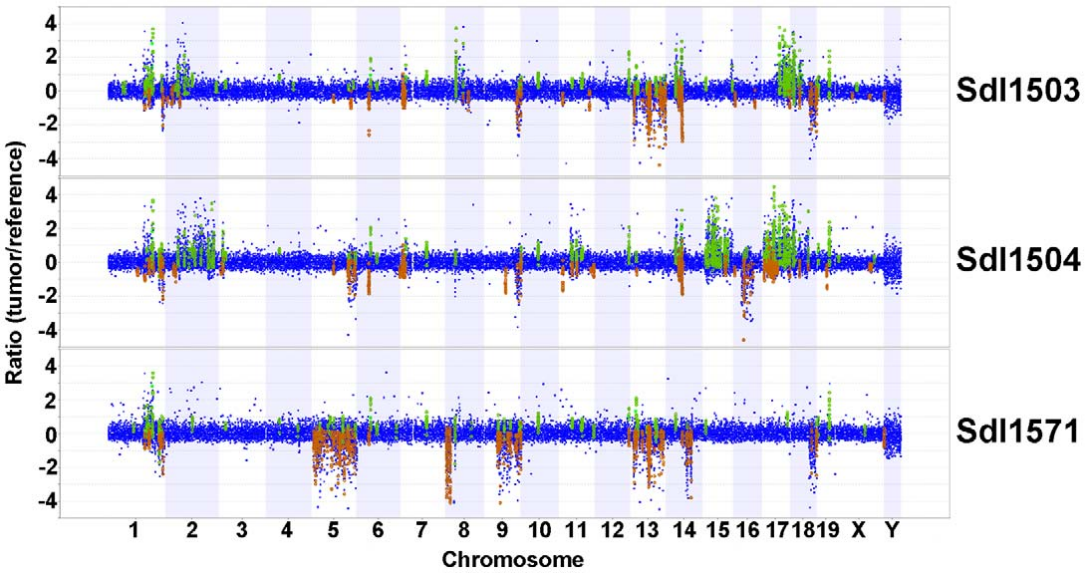
Array CGH profiles of thymic tumors from three *Sdl* mice. Probes with copy number gains and losses in tumors compared to reference are shown in green and red, respectively.

To determine if *Sdl* T-ALLs harbor additional genomic aberrations, array CGH was performed on genomic DNA isolated from *Sdl* thymic tumors compared to DNA isolated from a non-carrier mouse. Although whole chromosome gains and losses were not detected, many small deletions and amplifications averaging 110 kb in size were present in tumors (Figure 6). Therefore, the *Sdl* mutation promotes focal copy number changes and not aneuploidy.

## Discussion

We have been studying a novel spontaneous mouse cancer model, *Sdl*, in which an early-onset T-ALL phenotype is inherited in a dominant manner. We have accumulated evidence that *Mcm4^D573H^* is the causative tumor-causing genetic lesion in this model. The dominant inheritance of the cancer phenotype observed in *Sdl* contrasts to previous studies of mice harboring *Mcm2* (*Mcm2^IRES-CreERT2^*) or *Mcm4* (*Mcm4^chaos3^*) hypomorphic alleles in which tumors were only observed in the homozygous state [3], [5]–[7]. *Mcm2^IRES-CreERT2/IRES-CreERT2^* and *Mcm4^chaos3/chaos3^* mice harbor reductions in MCM levels detectable by Western analysis, which leads to a loss of backup origins that normally maintain genomic instability by firing during times of replicative stress [3]–[7]. Gene trap (GT) null alleles of *Mcm2* and *Mcm4* have also been generated. *Mcm2^GT/+^* mice have been reported to develop tumors, but only after one year of age and with approximately 75% penetrance [4]. Therefore, it has been proposed that a threshold level of MCM proteins (between 35 and 50% of normal for MCM2) is required for sufficient origin licensing to maintain genomic stability and prevent tumor formation [4]. We did not detect a reduction in total or chromatin bound MCM levels in *Sdl* mice, suggesting that the *Mcm4^D573H^* allele acts in a different mechanism to cause tumorigenesis. However, it is also possible that *Mcm4^D573H^* mice harbor small reductions in MCM levels that are beyond the detection limits of Western analysis. Although detailed aging studies were not presented, *Mcm4^GT/+^* mice were reported to be apparently normal [3]. However, a thorough study of the tumor phenotype of *Mcm4^GT/+^* mice will be required to determine the threshold levels of active MCM4 that are required to maintain genomic stability and prevent tumorigenesis.

The tumor spectrum and latency in *Sdl* is also very different from that observed in *Mcm4^chaos3^* ^/*chaos3*^ mice [3], [6]. The reasons underlying these differences remain to be elucidated. As *Mcm4^chaos3^* ^/*chaos3*^ and *Sdl* have been studied on different strain backgrounds, genetic modifiers could contribute to the observed phenotypic differences. Alternatively, the recessively acting hypomorphic *Mcm4^chaos3^* and the dominantly acting *Mcm4^D573H^* may have different consequences on origin licensing and DNA replication. In addition, a recent report found that MCM 2, 3, 5 and 7 regulate HIF1 activity and this function is likely independent from their function in the heterohexamer. A similar activity was not detected for MCM4 or 6 [18]. Therefore, the reduction in levels of total MCMs seen in *Mcm4^chaos3/chaos3^* mice could also influence HIF1 activity and have phenotypic consequences. A study of tumor and DNA replication phenotypes for both alleles on the same genetic background will be required to address the reasons for phenotypic differences between the two alleles.

Analysis of T cell differentiation in *Sdl* carriers revealed subtle defects, with some animals being more severely affected than others. One potential interpretation is that T cell differentiation is mostly normal in *Sdl* mice until genomic mutations due to replicative stress start to accumulate. In support of this, microarray data failed to detect any transcripts that are significantly differentially expressed in *Sdl* carrier thymuses compared to wild-type thymuses. Tests for common function did identify differences in genes with common gene functions including protein localization or targeting to mitochondria, chemokine binding or receptor activity and endothelial cell proliferation.

In contrast, many expression differences were detected between wild-type thymuses and *Sdl* leukemias. Many of the mostly profoundly down-regulated genes in *Sdl* leukemias are genes such as *Prss16* and *Tbata* that are expressed in thymic epithelial cells [19], [20]. This observation likely results from a lower ratio of T cells to thymic epithelial cells in normal thymus than in thymic lymphoma. Although non-T lineage cells are the minority of cells in the developing thymus, they nevertheless impacted our ability to identify genes that are down-regulated during T-ALL formation in *Sdl* mice. The transcripts with the greatest fold up-regulation in *Sdl* leukemias compared to normal thymus include genes with unknown function, metabolic genes, genes expressed during T cell activation and *Notch1* target genes. RT-PCR in *Sdl* tumors demonstrated the presence of an aberrant *Notch1* transcript splicing from exon 1 to exon 28 in 12 of 15 *Sdl* leukemias. Genomic PCR on a separate cohort of *Sdl* T-ALLs was able to clone genomic breakpoints in the *Notch1* locus in 3 of 13 tumors. These breakpoints occurred in introns 2 and 27, introns 1 and 27, and introns 2 and 26. It is possible that the exon 1 to 28 splice is favored even when deletions leave more internal exons intact, or that our RT-PCR conditions failed to robustly amplify transcripts containing other aberrant splice variants. Alternatively, the genomic re-arrangements present at the *Notch 1* locus may be more complex than can be detected by our genomic PCR. Nevertheless, the detected *Notch1* transcript and lack of RSS-like sequences at the cloned breakpoints are both consistent with the presence of type 2 deletions at the *Notch1* locus in *Sdl* T-ALLs. The vast majority of murine T-ALLs previously examined have harbored type 1 RAG-mediated deletions, while type 2 deletions were more rare. A predisposition to T-ALL has also been observed for *Mcm2* hypomorphic mice [5], [7] and array CHG detected deletions at the *Notch1* locus in 4 of 8 of T-ALLs in *Mcm2* mice [21]. One possibility to explain the tumor spectrum in *Sdl* mice and *Mcm2* hypomorphic mice is that the integrity of the murine *Notch1* locus is sensitive to replicative dysfunction in developing T cells and that replicative stress promotes the formation of type 2 deletions at the *Notch1* locus. As the majority of T cell development is completed by young adulthood, *Mcm4^chaos3/chaos3^* mice may not experience sufficient replicative stress to cause *Notch1* deletions in developing thymocytes, which would allow them survive longer to develop other late-onset tumor types.

Previous array CGH studies of *Notch1*-driven mouse T-ALLs failed to detect tumor-specific chromosomal aberrations, indicating that chromosomal instability is not a general characteristic of mouse T-ALL [22]. In contrast, array CGH data of *Sdl* tumors did detect small amplifications and deletions but not whole chromosome gains and losses. This data is consistent with previous observations that an improved growth phenotype found in *mcm4^Chaos3/Chaos3^* diploid yeast is due to mutations in a few genes and not due to aneuploidy [23]. In addition, recent array CGH experiments on T-ALLs from *Mcm2* hypomorphic mice also detected small genomic aberrations [21]. However, aberrations in T-ALLs in *Mcm2* mice were primarily deletions, while both amplifications and deletions were found in *Sdl* T-ALLs. It is possible that functional differences between MCM helicase activity in *Sdl* and *Mcm2* hypomorphic mice could explain this difference. However, it is also possible that strain specific modifiers can impact the types of aberrations generated by replicative dysfunction or selected for during tumorigenesis. Nevertheless, studies in yeast, *Mcm2* hypomorphic mice and *Sdl* mice all support a model that replicative stress can contribute to tumorigenesis by generating smaller chromosomal aberrations and not by causing aneuploidy.

The residue impacted by the observed *Mcm4* mutation in *Sdl* mice is part of the Walker B box, one of the structural motifs in MCM4 that is an integral part of the ATPase active site formed between MCM4 and MCM7 in the heterohexameric complex [24]. Engineering the D to H mutation into the analogous residue in yeast *mcm4* failed to complement a *mcm4* genomic deletion. Previous studies in yeast where the analogous D residue was mutated to A or T did complement a *mcm4* deletion allele [9]. Mutation of the D and the adjacent E residue to N and Q, respectively, (DE>NQ) did however fail to complement [25]. As the D residue in the Walker B box is believed to be important for coordinating the Mg^2+^ ion involved in ATP hydrolysis [26], the substitution of a positively charged H residue could result in a greater impact on MCM4 function than would mutation to an A or T (Figure S5). Given the observation that total and chromatin bound MCM levels are not different in *Sdl* carrier and wild-type mice, this supports a model in which MCM4^D573H^ containing helicases are stable, yet functionally inactive.

The role of MCM proteins in promoting genomic instability during human cancer initiation and progression remains unclear. Immunohistochemistry detects MCM protein expression in many human tumor samples, as would be expected for rapidly dividing cells [27]. Knockdown of *MCM 2, 3* or *7* in medulloblastoma cell lines caused inhibition of anchorage-dependent and independent growth; while their over-expression promoted cell migration, invasion and increased anchorage-independent growth [28]. *MCM7* over-expression in epithelial progenitor cells sensitized mice to carcinogen-induced skin tumors but did not itself drive tumors by 1 year of age [29]. Over-expression of *MCM7* alone in the prostatic epithelium did not promote phenotypes. However, over-expression of *MCM7* along with a *PTEN*-targeting microRNA cluster encoded within the *MCM7* human locus did initiate prostate tumorigenesis [30]. Although mutations in genes involved in DNA damage checkpoints and DNA damage repair are known to contribute to sporadic and hereditary tumorigenesis, it is unclear if genetic changes in the actual components of the replication machinery such as MCM proteins contribute to tumorigenesis in humans. A few point mutations in *MCM* subunits have been detected in human tumors [31]. However, the functional consequences of these mutations are currently unknown. Given clinical use and preclinical development of compounds that impact replication as cancer chemotherapies, it will be important to elucidate how *MCM*s contribute to tumor initiation and progression. Although previous studies have uncovered a tumor suppressive activity for *Mcm*s, our studies of the *Sdl* model indicate that dominantly acting *Mcm*s alleles can be compatible with viability but cause chromosomal abnormalities and highly penetrant tumor formation. Therefore, *Mcm* mutations with different functional consequences on MCM levels and activity have the potential to act as driver mutations during tumorigenesis.

## Materials and Methods

### Ethics statement

Mouse experiments were performed according to the institutional guidelines for animal care under the approval of the IACUC of the University of Minnesota and the University of Wisconsin.

### Animals

*Sdl* arose in the germline of a *Rosa-SB11* mouse maintained on the C57Bl/6 background [32]. Wild-type mice were purchased from Jackson Labs or Charles River. Non-carrier sibling mice were utilized as controls. *Mcm4^chaos3^* mice on the FVB/N genetic background were generously provided by Naoko Shima.

### DNA capture and exon re-sequencing

The SureSelect XT Mouse All Exon Kit (Agilent Technologies) was used to capture exonic sequences from genomic DNA purified from a tail clip of a *Sdl* carrier. Sequencing was performed on the Illumina Genome Analyzer 2 platform as paired-end 76-bp reads. One lane of sequence was generated. Reads were aligned to the MM9 reference genome with BWA v0.5.5 [33]. The GATK (v1.0.4771) [34] was then used to do local realignment of the reads around all indel sites called in the mouse genomes project [35]. The base qualities of the BAM file were recalibrated with the GATK v1.0.4771 by masking all SNP and indel positions called in the mouse genomes project. SNP calling was carried out using SAMtools mpileup/bcftools (v0.1.16) [36]. For SAMtools mpileup, the following options were used: -d 500 -C50 -m3 -F0.002 -aug. The raw sequence data is available under ERA accession number ERP000474.

### Array CGH

DNA was purified from thymic tumors from three *Sdl* mice on the 129S1/SvImJ genetic background. Tail clip DNA from a non-carrier also on the 129S1/SvImJ genetic background was utilized as reference DNA. Hybridizations were performed by WiCell research institute according to manufacturer's recommendations to the mouse CGH 3×720 K Whole-Genome Tiling Arrays (NimbleGen). NimbleScan, CGH Fusion (RBS v1.0) (Infoquant) software was utilized for analysis and data visualization. Gains and losses were called with the following parameters: average log-ratio threshold of 0.2, a minimum aberration length of 5 probes and maximum p-value of 0.001.

### Micronucleus assay

The micronucleus assay was performed essentially as described [37]. 5 week-old females on the FVB/N genetic background were analyzed.

### Western analysis

Whole cell protein extracts were purified using RIPA buffer and protease inhibitors (Pierce). Cell fractionation was performed using the Qproteome Nuclear Protein Kit (Qiagen). Proteins were run on a 10% SDS-polyacrylamide gel and transferred to a PVDF membrane. Membranes were incubated with primary antibodies, followed by incubation with the secondary antibody and visualized using Western Lighting Plus-ECL (PerkinElmer).

### RT–PCR and quantitative RT–PCR (qPCR)

RNA was isolated by Trizol (Invitrogen) and further purified using the RNeasy Mini Kit (Qiagen). All wild-type and *Sdl* carrier thymuses used for RNA extraction were from 21-day-old mice. Wild-type thymuses were isolated from non-carrier littermates. First strand cDNA was generated using a 20∶80 mix of polyT:random decamer primers (Ambion Retroscript). Real-time PCR was completed using the LightCycler (Roche Applied Science). Briefly, LightCycler FastStart DNA MasterPLUS SYBR Green I kit (Roche Applied Science) was used with 5 µl cDNA from the reverse transcriptase reaction. Relative mRNA expression levels were calculated as described previously [38]. Go taq (Promega) was used for non-quantitative PCRs. t-tests or ANOVA followed by Tukey post hoc analysis were performed using Prism software.

### Southern analysis

DNA was purified from thymic tumors, and TCR β re-arrangement was detected as described [8].

### Microarray analysis

RNA was isolated from female mice by Trizol (Invitrogen) extraction followed by further purification using the RNeasy kit (Qiagen). RNA from three animals for each group (21-day-old wild-type thymus, 21-day-old *Sdl* carrier thymus and *Sdl* overt thymic tumors) was pooled per array. Four RNA pools were analyzed per group. Amplified sense-strand cDNA was generated using the Ambion WT expression kit (Applied Biosciences), fragmented and labeled with the GeneChip WT Terminal Labeling Kit (Affymetrix) and hybridized to GeneChip Mouse Exon 1.0 ST Arrays (Affymetrix) by the University of Wisconsin Biotechnology Center Gene Expression Center. The data were normalized using *rma* [39] as implemented in the *xps* system, available at Bioconductor (www.bioconductor.org). Summaries at the transcript level using probes given a core level ranking were analyzed, as this annotation level is most conservative [40]. Antigenomic probes were used for background correction. EBarrays, an empirical Bayes hierarchical modeling approach, was used to identify differentially expressed (DE) genes [41], [42]. With EBarrays, a gene was considered significantly DE if the posterior probability of DE under the log-normal Normal moderated variance (PP.LNNMV.DE) model exceeded 0.95, as this threshold controls the posterior expected false discovery rate (FDR) at 5% [42]. We also considered results from gene specific t-tests with q-values calculated from the t-test p-values [43]. A list of genes with q-values <0.05 has expected FDR of 5%. To help ensure that results were robust to the statistical method used, we define a gene to be significantly DE if its PP.LNNMV.DE>0.95 and its q-value <0.05. Tests for enrichment of common function among sets of differentially-expressed genes were carried out using data from the Gene Ontology (GO) annotations and the Kyoto Encyclopedia of Genes and Genomes (KEGG). The R package allez was used to perform tests of enrichment for each GO category and KEGG pathway [44]. In general, the interpretation of p-values resulting from enrichment tests is not straightforward due to the many dependent hypotheses tested. Furthermore, the enrichment test tends to result in small p-values when groups with few transcripts are considered. The statistical methods underlying allez adjust for these factors, allowing increased power and sensitivity for identifying sets that are biologically meaningful.

### Yeast studies

The *mcm4* deletion strain and TRP1 plasmids were described previously [9] and were generously provided by Anthony Schwacha. The *Sdl* mutation in the equivalent residue (D632H) was generated by site-directed mutagenesis utilizing PfuUltra Hotstart taq (Agilent) followed by DpnI digestion and verified by sequencing. Cloning into the TRP1 plasmid added a HA/H10X tag that allowed Mcm4 D632H protein expression to be verified by Western blot. The engineered *Sdl* mutation creates a restriction fragment polymorphism that can be detected by PCR amplification followed by BssSI digestion.

### MEF isolation and analysis

MEFs were isolated from 12.5–15.5 dpc embryos on the 129S1/SvImJ genetic background. All MEFs were utilized at p3 or lower. For chromosome break analysis, MEFs were treated with aphidicolin or DMSO as vehicle only control for 24 hours. Colcemid was added for the final five hours prior to harvest. Three separate cultures were analyzed per genotype. Harvested cells were pelleted and re-suspended in 0.075 M KCl solution and incubated at room temperature for 20 minutes, pelleted, and re-suspended in 3∶1 (vol/vol) methanol/acetic acid for 10 minutes twice. Cells were spread on a slide and stained with Giemsa stain (Sigma GS500) in a 1∶20 dilution in water. Slides were washed briefly with 1× PBS and observed.

### Histopathology

Tissues were fixed in 10% formalin, embedded in paraffin, sectioned and stained with hematoxylin and eosin by the University of Wisconsin RARC comparative pathology laboratory. Analysis was performed by the UWCCC Experimental Pathology Core.

### Flow cytometry

Cryopreserved tumor cell samples were briefly thawed at 37°C and then incubated for 15 min at 37°C in DMEM containing 20% FBS, 10 U/ml Heparin (Sigma), and 0.25 mg/ml DNAse 1 (Roche). Cells were pelleted and re-suspended in HBSS without Ca/Mg, 2% FBS, 2.5% cell dissociation buffer (GIBCO, Invitrogen), 100 U/ml Penicillin G, and 100 µg/ml streptomycin. Tumor cell suspensions were pre-incubated with antibodies to FcγRII/III (BD PharMingen) for 3 min to prevent nonspecific binding of labeled antibodies to the cell surface. They were then stained with monoclonal antibodies conjugated with biotin, phycoerythrin (PE), or TRI-COLOR (TC) for 20 min at 4°C. After washing, strepavidin-allophycocyanin (APC) was added for 20 min at 4°C to provide fluorescent signal for biotinylated antibodies. Staining was assessed with a FACSCalibur flow cytometer (Becton Dickinson) in four-color mode using CellQuest Pro and FlowJo software for analysis. For thymocyte analysis, dissociated cells were incubated with anti-CD4 FITC and anti-CD8 APC to examine CD4, CD8 and CD4/8 double positive T cells. To examine double negative (DN) populations, dissociated thymocytes were stained with a lineage cocktail (anti Gr-1-Biotin, γδTCR-Biotin, B220-Biotin, NK1.1-Biotin, TER-119-Biotin, Streptavidin FITC, CD4-FITC, Mac-1 FITC, CD8-FITC) along with CD25-PE, CD44-AlexaFluor700 and DAPI. DN thymocytes were defined as the FITC negative gate. At least 10,000 DN cells per animal were analyzed for CD25 and CD44 expression. DN1 cells were defined as Lin− CD25− CD44+, DN2 cells as Lin− CD25+ CD44+, DN3 cells as Lin− CD25+ CD44− and DN4 cells as Lin− CD25− CD44−. All animals utilized for analysis of thymocyte developmental stages were female progeny of one *Sdl* mouse on the FVB/N genetic background.

Details of exon capture methods, antibodies utilized and primer sequences for genotyping and RT-PCR are provided as Text S1.

## Supporting Information

Figure S1 *Sdl* mice primarily develop T-ALL. A) H&E stain of bone marrow in the sternum of a *Sdl* mouse showing the presence of neoplastic cells (40× magnification, scale bar = 50 µM) B) H&E stain of a kidney from a *nude/nude* mouse that received T-ALL cells from a *Sdl* mouse showing infiltration of transplanted neoplastic lymphocytes (40× magnification, scale bar = 50 µM). C) Southern analysis of *Sdl* tumors detects re-arrangements at the TCR β locus. Asterisk indicates germline band, arrowhead indicates an example of a re-arrangement. D) Western analysis detects TdT expression in *Sdl* leukemias (top). Tubulin is used to demonstrate equal protein loading (bottom).
(TIF)

Figure S2 Inter-animal differences in T cell developmental defects in *Sdl* carriers. CD44 and CD25 staining of Lin- thymocytes was used to examine DN stages of T cell development in four non-carrier (wild-type, WT) and four Sdl carrier (C) thymuses. Differences in the percent of cells at the DN1 (CD25− CD44+) stage of development were statistically significantly decreased in *Sdl* carriers compared to wild-type (See Table 2), with animals C3 and C4 more severely affected than animals C1 and C2. Animals C3 and C4 also appear to have a defect at the DN3 (CD25+ CD44−) to DN4 (CD25− CD44−) transition.
(TIF)

Figure S3 qRT-PCR detects no differences in expression levels of genes in the *Sdl* interval between wild-type (solid bars) and carrier thymuses (striped bars). Wild-type thymus is normalized to 1. N = 3 (except for *Mcm4* carrier thymus where N = 6), error bars indicate standard deviation. P values are all >0.29. Expression of F830005K03Rik was not detected. *Mcm4* data is also depicted in Figure 3.
(TIF)

Figure S4 Histogram showing sequencing depth coverage of exons in the *Sdl* interval. The number of base pairs (frequency) is plotted against sequencing depth coverage.
(TIF)

Figure S5 A) Alignment of MCMs around the residue equivalent to murine MCM4 573 D (indicated in bold). Murine and yeast MCM 2–7 are shown, as are MCM4 from several eukaryotes. The residue impacted by the *Sdl* mutation is invariant across MCMs. B–D) The structure of an archaeal (*Sulfolobus solfataricus*) MCM helicase [45] with the equivalent D residue highlighted (B) was utilized to model the impact of the D to H *Sdl* mutation (C) and the D to A mutation (D) that was previously shown in complementation tests to not impact the biologic activity of *S. cerevisiae mcm4* [9]. The D to H substitution is predicted to have a greater impact on protein structure than the D to A substitution. Structures were visualized using PyMol [46].
(TIF)

Figure S6 Total and chromatin bound levels of MCM4 are similar in *Sdl* and wild-type MEFs. Western analysis on total MEF protein extract as well as purified chromatin bound (c.b.) fractions indicate that *Sdl* carrier MEFs (C) harbor similar levels of MCM4 protein as do wild-type (WT) MEFs. TUBULIN and Ponceau S membrane staining were utilized to demonstrate equal loading for whole cell lysates and chromatin bound fractions, respectively.
(TIF)

Table S1 Summary of flow data for Sdl leukemias. Mouse 1 corresponds to panel D, Mouse 2 to panel E, Mouse 3 to panel F and Mouse 4 to panel G in Figure 1. LN = lymph node, SPL = spleen, THY = thymus. + positive staining. − no staining.
(XLSX)

Table S2 Summary of exon and splice site sequences with fewer than 10× coverage by exon-capture re-sequencing that were examined by PCR amplification followed by Sanger sequencing.
(XLSX)

Table S3 Non-synonymous changes identified by exon capture on proximal chromosome 16. * The change in *Top3b* falls outside of the *Sdl* interval (see Figure 2A) and was not confirmed by traditional Sanger sequencing in genomic DNA from the mouse utilized for exon capture or other carrier *Sdl* mice. Visual inspection indicates that the base lies adjacent to a low-complexity polyG tract and that the alternate call is likely due to read misalignment.
(XLSX)

Text S1 Supplemental methods.
(DOC)
