# Supplementary figures and images for: A Dominantly Acting Murine Allele of Mcm4 Causes Chromosomal Abnormalities and Promotes Tumorigenesis
Source: PLoS Genet. 2012 Nov 1;8(11):e1003034. doi: 10.1371/journal.pgen.1003034 (PMC3486839; doi:10.1371/journal.pgen.1003034)

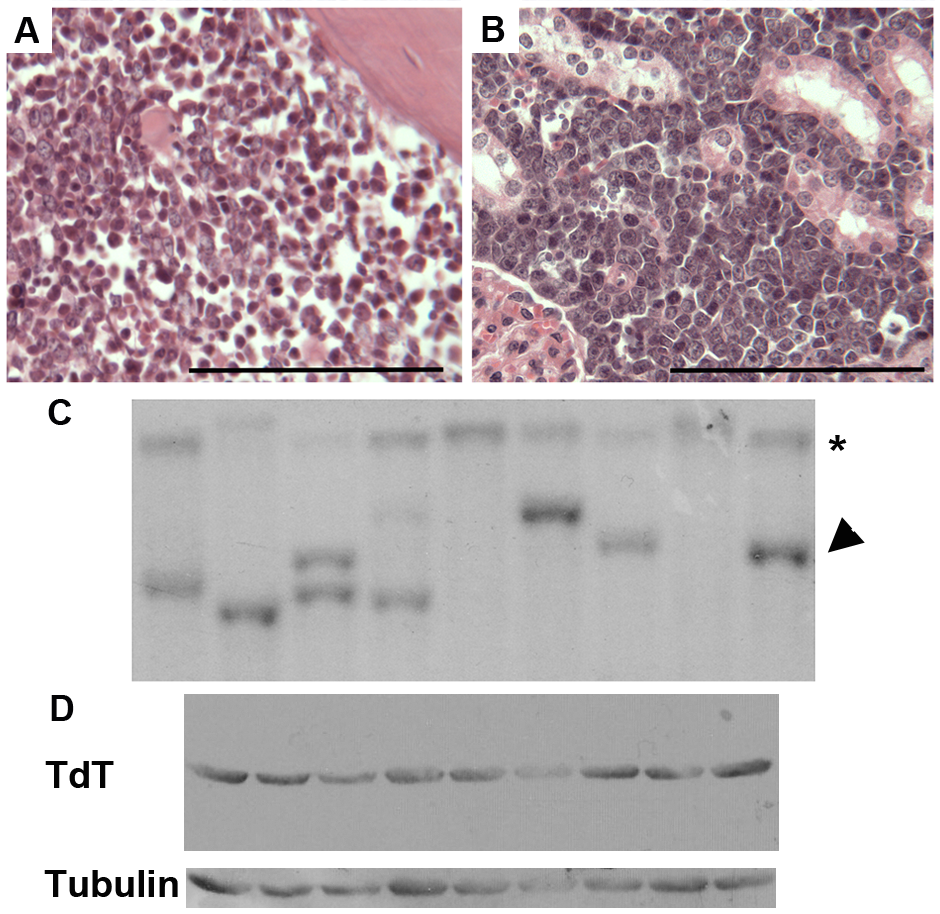

Supplement: Figure S1 — Sdl mice primarily develop T-ALL. A) H&E stain of bone marrow in the sternum of a Sdl mouse showing the presence of neoplastic cells (40× magnification, scale bar = 50 µM) B) H&E stain of a kidney from a nude/nude mouse that received T-ALL cells from a Sdl mouse showing infiltration of transplanted neoplastic lymphocytes (40× magnification, scale bar = 50 µM). C) Southern analysis of Sdl tumors detects re-arrangements at the TCR β locus. Asterisk indicates germline band, arrowhead indicates an example of a re-arrangement. D) Western analysis detects TdT expression in Sdl leukemias (top). Tubulin is used to demonstrate equal protein loading (bottom). (TIF) [file pgen.1003034.s001.tif]

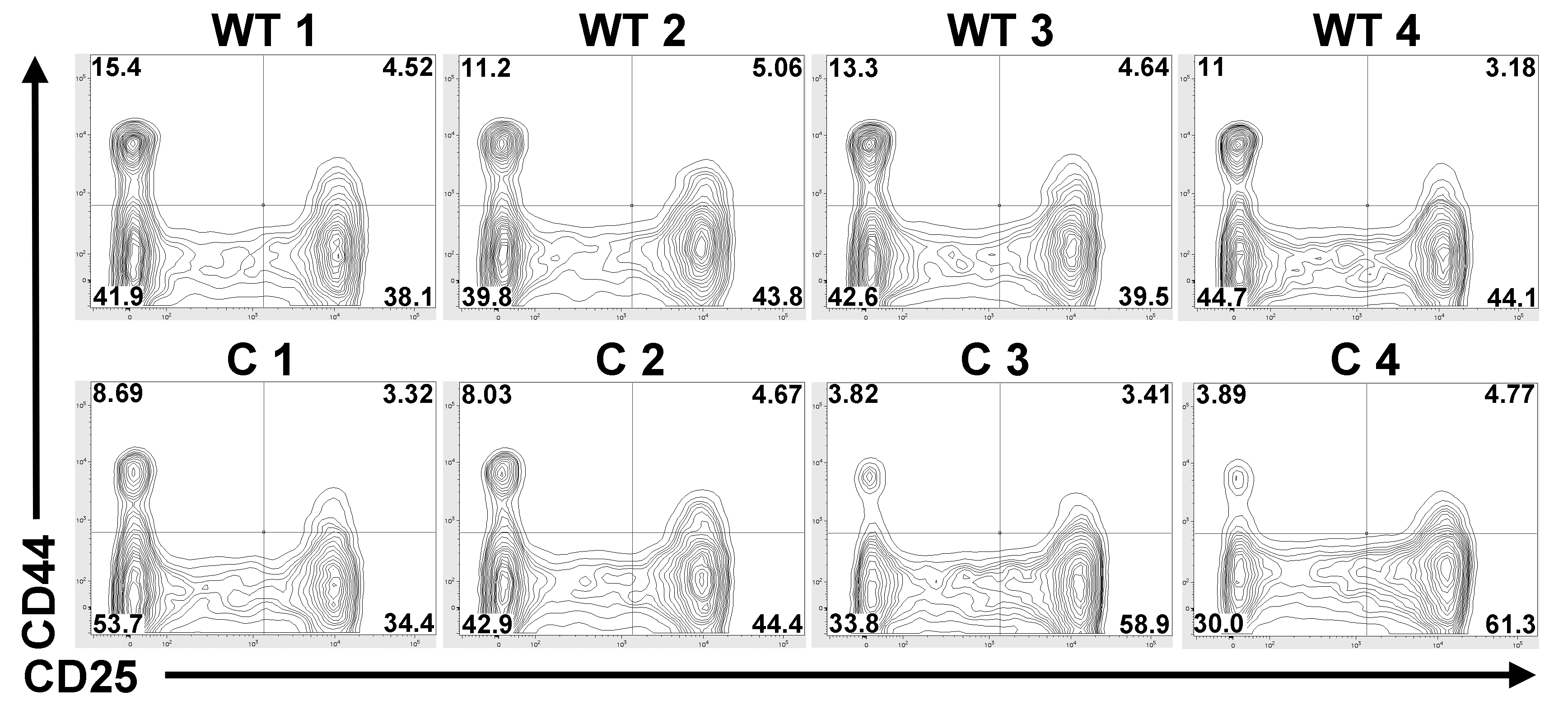

Supplement: Figure S2 — Inter-animal differences in T cell developmental defects in Sdl carriers. CD44 and CD25 staining of Lin- thymocytes was used to examine DN stages of T cell development in four non-carrier (wild-type, WT) and four Sdl carrier (C) thymuses. Differences in the percent of cells at the DN1 (CD25− CD44+) stage of development were statistically significantly decreased in Sdl carriers compared to wild-type (See Table 2), with animals C3 and C4 more severely affected than animals C1 and C2. Animals C3 and C4 also appear to have a defect at the DN3 (CD25+ CD44−) to DN4 (CD25− CD44−) transition. (TIF) [file pgen.1003034.s002.tif]

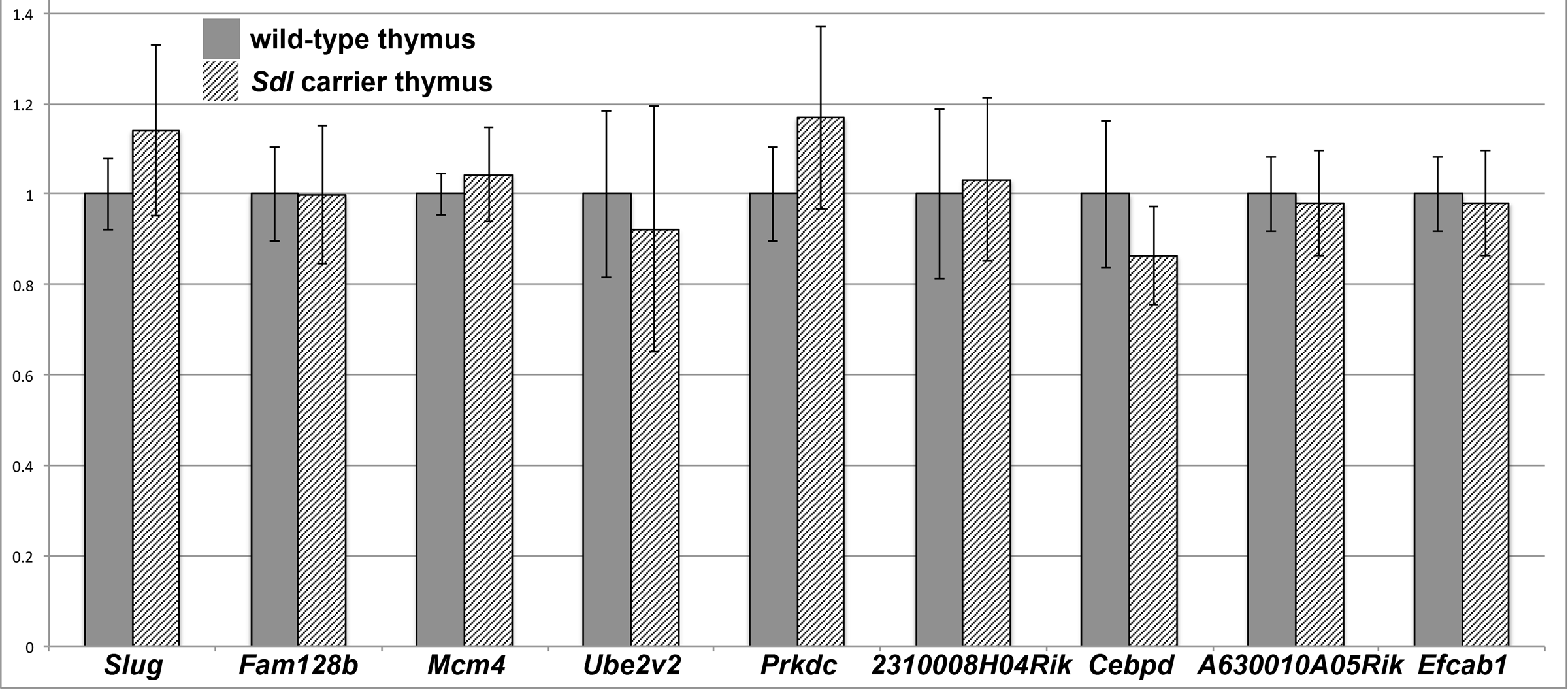

Supplement: Figure S3 — qRT-PCR detects no differences in expression levels of genes in the Sdl interval between wild-type (solid bars) and carrier thymuses (striped bars). Wild-type thymus is normalized to 1. N = 3 (except for Mcm4 carrier thymus where N = 6), error bars indicate standard deviation. P values are all >0.29. Expression of F830005K03Rik was not detected. Mcm4 data is also depicted in Figure 3. (TIF) [file pgen.1003034.s003.tif]

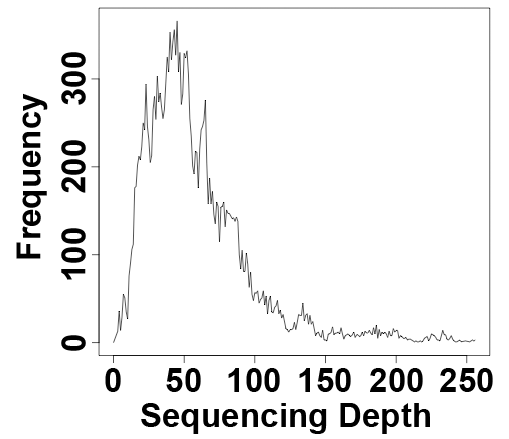

Supplement: Figure S4 — Histogram showing sequencing depth coverage of exons in the Sdl interval. The number of base pairs (frequency) is plotted against sequencing depth coverage. (TIF) [file pgen.1003034.s004.tif]

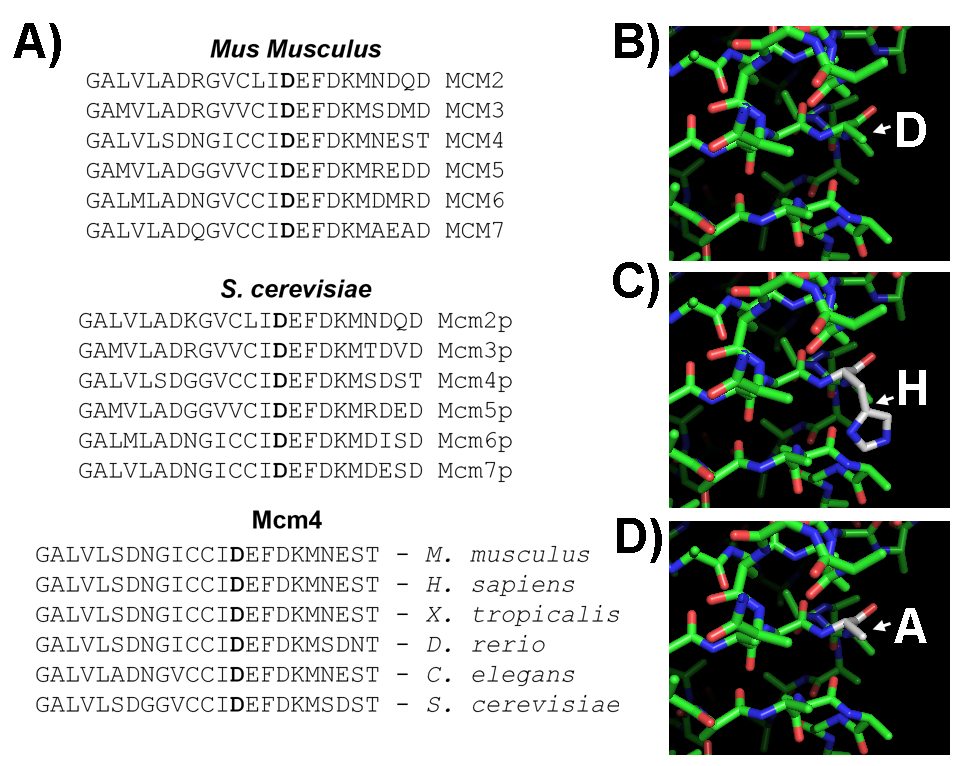

Supplement: Figure S5 — A) Alignment of MCMs around the residue equivalent to murine MCM4 573 D (indicated in bold). Murine and yeast MCM 2–7 are shown, as are MCM4 from several eukaryotes. The residue impacted by the Sdl mutation is invariant across MCMs. B–D) The structure of an archaeal (Sulfolobus solfataricus) MCM helicase [45] with the equivalent D residue highlighted (B) was utilized to model the impact of the D to H Sdl mutation (C) and the D to A mutation (D) that was previously shown in complementation tests to not impact the biologic activity of S. cerevisiae mcm4 [9]. The D to H substitution is predicted to have a greater impact on protein structure than the D to A substitution. Structures were visualized using PyMol [46]. (TIF) [file pgen.1003034.s005.tif]

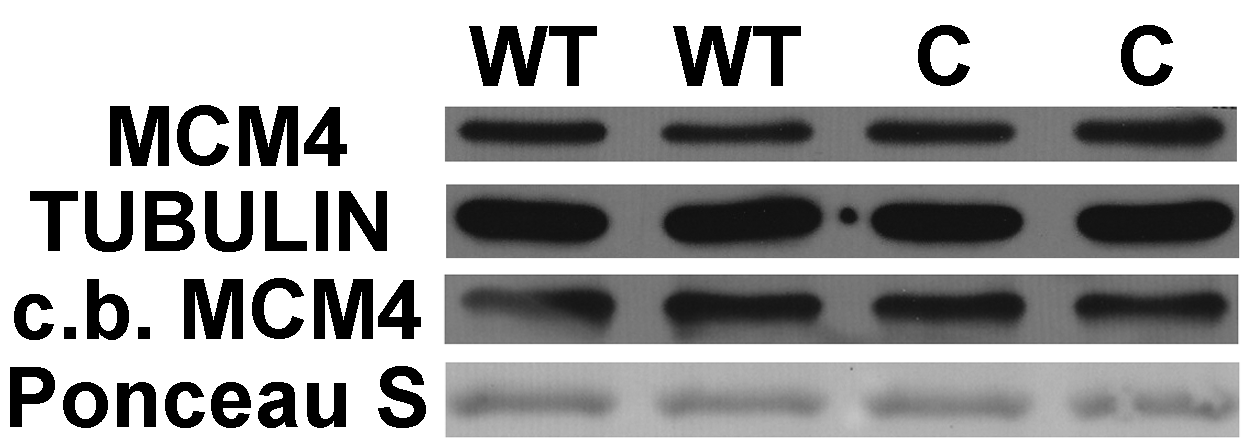

Supplement: Figure S6 — Total and chromatin bound levels of MCM4 are similar in Sdl and wild-type MEFs. Western analysis on total MEF protein extract as well as purified chromatin bound (c.b.) fractions indicate that Sdl carrier MEFs (C) harbor similar levels of MCM4 protein as do wild-type (WT) MEFs. TUBULIN and Ponceau S membrane staining were utilized to demonstrate equal loading for whole cell lysates and chromatin bound fractions, respectively. (TIF) [file pgen.1003034.s006.tif]
