# Supplementary material for: A Dominantly Acting Murine Allele of Mcm4 Causes Chromosomal Abnormalities and Promotes Tumorigenesis
Source: PLoS Genet. 2012 Nov 1;8(11):e1003034. doi: 10.1371/journal.pgen.1003034 (PMC3486839; doi:10.1371/journal.pgen.1003034)
Supplement: Text S1 — Supplemental methods. (DOC) [file pgen.1003034.s010.doc]

| **Supplemental methods:**  Details of exon capture:  (2-3 μg) of genomic DNA was sheared to 100–400 bp using a Covaris E210.  Sheared DNA was subjected to Illumina paired-end DNA library preparation (NEBNext DNA Sample Prep Set 1; New England BioLabs) and the adapter-ligated library was amplified for 5-6 cycles using Herculase II (Agilent Technologies) with PE1.0 and PE2.0 oligonucleotides (Illumina).  Amplified library (500 ng) was hybridized to a mouse bait library (SureSelect XT Mouse All Exon Kit; Agilent Technologies).  Hybridized material was captured using streptavidin-coated beads (Invitrogen), was amplified for 10-11 cycles using Herculase II with PE1.0 and PE2.0 oligonucleotides (Illumina) prior to sequencing.  Antibodies Utilized:  For Westerns, primary anitbodies were from Abcam: anti-TdT (ab79395), anti-Mcm2 (ab4461), anti-Mcm4 (ab4459, ab84153), and anti-β-tubulin (ab6046). Abcam antibody ab16284 was used as a secondary antibody. For tumor flow cytometry, cell were stained with antibodies to CD3, CD4, CD5, CD8a, CD11b (Mac1), CD19, CD45, CD45R (B220), CD71, CD90.2, CD117 (Kit), IgM, Ly-6G (Gr1), Ly-71 (F4/80), and Ly-76 (Ter119). Most antibodies were purchased from BD PharMingen, whereas CD11b TC, F4/80 PE, CD19 TC, CD45 TC, and CD4 TC were from CalTag. For thymocyte analysis antibodies utilized were: anti-CD4 FITC clone GK1.5, (eBioscience), anti-CD8 APC clone 53-6.7 (BDbioscience), anti Gr-1-Biotin clone RB6-8C5 (eBioscience), γδTCR-Biotin clone eBioGL3 (eBioscience), B220-Biotin clone RA3-6B2 (eBioscience), NK1.1-Biotin clone-PK136 (eBioscience), TER-119-Biotin (eBioscience), Mac-1 FITC clone-M1/70 (BDbioscience), CD8-FITC clone-53-6.7 (BDbioscience), CD25-PE clone-7D4 (BDbioscience) and CD44-AlexaFluor700 clone-IM7 (BDbioscience). Streptavidin FITC (eBioscience) was used for biotinylated antibodies.  Primers utilized: | |
| --- | --- |
| SSLPs and SNPs: |  |
| rs4165069 fwd | 5- GCC ACC CTG AAG AAA GTC AC -3 |
| rs4165069 rev | 5- ACC CCC TCC CTC TAC ACA TC -3 |
| D16Mit4 fwd | 5- AGT TCC AGG CTA CTT GGG GT -3 |
| D16Mit4 rev | 5- GAG CCC TCA TTG CAA ATC AT -3 |
| D16Mit131 fwd | 5- TGG TGG TGG TGT TGA TGG TA -3 |
| D16Mit131 rev | 5- AAG ACC ATT TCT AAT AAA CAA CAC CC -3 |
| Mcm4 Sdl fwd | 5- AAA TTC CGT GCT GAG ATC AA -3 |
| Mcm4 Sdl rev | 5- TTC CAT GAC CTC ATG CAG CA -3 |
| D16LC1 fwd | 5- AGT TGT ATT ATTTGC ATC TCC AAC C -3 |
| D16LC1 rev | 5- ACA AGT TCC AAG CCA TTC AG -3 |
| rs4164765 fwd | 5- AGG CCC AGA GTA TCC AAA TTA ACA -3 |
| rs4164765 rev | 5- ACT AAA GCT ATC TGA CCC TGG GCT -3 |
| rs4164987 fwd | 5- TAA ACT GAA GAG CCC TGG TGT CCT -3 |
| rs4164987 rev | 5- TGT GAG CTT GTC AGA TCC TCT GGA -3 |
| Sequencing Notch1: |  |
| Notch1 exon 26 gen fwd | 5- CAT TGC GAG TCG CCA AGC AC -3 |
| Notch1 exon 26 gen rev | 5- GTT AGA GAA CCC AGG GAC ACA CTG -3 |
| Notch1 exon 27 gen fwd | 5- GGG CAA GGC TAG ACA GTG GA -3 |
| Notch1 exon 27 gen rev | 5- TCA GGA CCC TGT GCA TGC CT -3 |
| Notch1 exon 34a gen fwd | 5- CCC CAG GCC ATC CAT CAT CA -3 |
| Notch1 exon 34a gen rev | 5- TGC CTG TGT GCT CAG TGT GC -3 |
| Notch1 exon 34b gen fwd | 5- CTC CAG AAT GGC ATG GTG CC -3 |
| Notch1 exon 34b gen rev | 5- CCT GAC CAG GAA AAT CAA GGC TC -3 |
| Notch1 exon 1 fwd | 5- AAA GAG GGC ATC AGA GGGTGG A -3 |
| Notch1 exon 2 fwd | 5- GTG GGA CCT GCC TGA ATG GAG -3 |
| Notch1 intron 2 a fwd | 5- CCC ATC CAT TCC ATG GTG TTG TCT -3 |
| Notch1 intron 2 b fwd | 5- AGA GCA GAC AGG AGA ACC GTG TTT -3 |
| Notch1 intron 2 c fwd | 5- TTT GGC CCT GTC TTC CAT AGG CTT -3 |
| Notch1 intron 2 d fwd | 5- TCC TCT AGC TTG CTC AGC AGT CAT -3 |
| Notch1 intron 2 e fwd | 5- TGC CTG TGA CTG TTC TCT GAA CCA -3 |
| Notch1 intron 2 f fwd | 5- TCA CCA CTT GTT GAG TGG GAG CTT -3 |
| Notch1 intron 2 g fwd | 5- AGT GTT GGC AAG TGT GCA TCC CAT -3 |
| Notch1 intron 3 h fwd | 5- AGC AGA CCA GCC TCC TAA ATG AGA -3 |
| Notch1 exon 27 rev | 5- ATG ACT GCA CAC ATT GCC GGT TGT -3 |
| Notch1 exon 28 rev | 5- TGC CAC GTA CAT GAG GTG CAG -3 |
| qPCR: |  |
| Notch1 exon 1 fwd | 5- AAA GAG GGC ATC AGA GGGTGG A -3 |
| Notch1 exon 24 fwd | 5- TGG ATT CAT CTG TAG GTG CCC TG -3 |
| Notch1 exon 25 rev | 5- ATG CAT GTA CCA CCG TTG AGG CA -3 |
| Notch1 exon 26 fwd | 5- CCA CCT CTT CAC TGC TTC CTG GTA -3 |
| Notch1 exon 27 rev | 5- ATG ACT GCA CAC ATT GCC GGT TGT -3 |
| Notch1 exon 27 fwd | 5- TTC CTA GGT GCT CTT GCG TCA CTT -3 |
| Notch1 exon 28 rev | 5- TGC CAC GTA CAT GAG GTG CAG -3 |
| Notch1 exon 28 fwd | 5- TGT CAG AGG CCA GCA AGA AGA A -3 |
| Notch1 exon 29 rev | 5- TGA TTG TCG TCC ATC AGA GCA CCA -3 |
| Notch1 exon 29 fwd | 5- ATG GAC GAC AAT CAG AAC GAG TGG -3 |
| Notch1 exon 30 rev | 5- AGG GAG AAC TAC TGG CTC CTC AAA -3 |
| Notch1 exon 30 fwd | 5- AGG TGG ATG CTG ACT GCA TGG AT -3 |
| Notch1 exon 31 rev | 5-TCC ACT GCA GGA GGC AAT CAT GAG G -3 |
| Notch1 exon 34 (3'UTR) fwd | 5- AGT GTG ACC CAG ACC TTG TGA -3 |
| Notch1 exon 34 (3'UTR) rev | 5- AGT GGC TGG AAA GGG ACT TG -3 |
| Myc fwd | 5- TTG AAG GCT GGA TTT CCT TTG GGC -3 |
| Myc rev | 5- TCG TCG CAG ATG AAA TAG GGC TGT -3 |
| Hes1 fwd | 5- AAA GCA TCA AAG CCT ATC AT -3 |
| Hes1 rev | 5- GTC TGC CTT CTC TAG CTT GG -3 |
| Mcm2 fwd | 5- AAT GCA GAC CTT TAC CTG TGA GCC -3 |
| Mcm2 rev | 5- CAG CCT TCT AAT GAG CCT TGG ACA -3 |
| Mcm3 fwd | 5- TGA CGA TTC CCA AGA GAA GAC CGA -3 |
| Mcm3 rev | 5- TTC CTG GAA CAC TTC TAA GAG GGC -3 |
| Mcm4 fwd | 5- CAC CAG CCT TAA AGT ACC AAC AGC -3 |
| Mcm4 rev | 5- AAA GCT CGC AGG GCT TCT TCA AAC -3 |
| Mcm5 fwd | 5- TTT GCC ATT GGC TCT CAG GTG TCT -3 |
| Mcm5 rev | 5- ATG AGC TGC AGC ACC TTT CGG ATA -3 |
| Mcm6 fwd | 5- TGA GGC TGG GCT TTG CTG AAT ACT -3 |
| Mcm6 rev | 5- AAG CTC GCT TCT CTT TAG TGC CGA -3 |
| Mcm7 fwd | 5- TGC TCT GCT TTC TCG ATT CGA CCT -3 |
| Mcm7 rev | 5- TGT GCT GGT GGA CAT AGG TGA TGT G -3 |
| Slug fwd | 5- CTT GTG TCT GCA AGA TCT GTG GCA -3 |
| Slug rev | 5- TGG AGC AGT TTT TGC ACT GGT AT -3 |
| Fam128b fwd | 5- GGA TTG GAG ACA GCA ACG CTG -3 |
| Fam128b rev | 5- AGC ATC TGG AAG ACG GCG AG -3 |
| Ube2v2 fwd | 5- TGT TGG AAG AAC TTG AAG AAG GAC A -3 |
| Ube2v2 rev | 5- TGG AGG AGC TTC TGG GTA TTT AGA T -3 |
| Prkdc fwd | 5- TGC ATC TCA ATT CAC TGC CTG C -3 |
| Prkdc rev | 5- AAG TAC TTC AGC CTG CTC TTA TGC A -3 |
| 2310008H04 fwd | 5- TTC CAG TGA AAG TGA TTT CTC CGA -3 |
| 2310008H04 rev | 5- TCC AGG GTA CCC TTA TCA TCT TCA -3 |
| Cebpd fwd | 5- ATG CAG CAG AAG CTG GTG GAG TT -3 |
| Cebpd rev | 5- TTT GAA GAA CTG CCG GAG GCA A -3 |
| A630010A05 fwd | 5- AAT CTG AAG CTT CTG ATG AAG AGC C -3 |
| A630010A05 rev | 5- TCA GTT GCT CAT GCC AGT -3 |
| Efcab1 fwd | 5- CTA TAA TTT GGT GGG AGA CGT AGC A -3 |
| Efcab1 rev | 5- CCC TCG AAG AAA CAG TGA TAA TCC A -3 |
| F830005K03 fwd | 5- CAG TGG TGG TGA GAC ATG GTC A -3 |
| F830005K03 rev | 5- CTG CAG AAG AAG AGT GAT TCA GCA -3 |
| Yeast Primers: |  |
| Site Directed Mutagenesis fwd | 5- GTC TGA TGG AGG TGT TTG TTG TAT TCA CGA GTT TG -3 |
| Site Directed Mutagenesis rev | 5- CAA ACT CGT GAA TAC AAC AAA CAC CTC CAT CAG AC -3 |
| Yeast Mcm4 fwd | 5- TCC GGG TAA AGG TTC ATC TGC CGT -3 |
| Yeast Mcm4 rev | 5- TGA ACC AAT TGG GTT ACC ACT GGC -3 |
| Sanger sequencing:  A630010A05Rik for | 5- GGG AGA ACC TTC ATA GGA GCA GGG -3 |
| A630010A05Rik rev | 5- CAC CAT CTG CAA TAC CGC AGA CAT -3 |
| Efcab1 1for | 5- GAT TCC TAC CAA TTA GGA TTA TGG CTA CAC -3 |
| Efcab1 1rev | 5- CAC TGA GCC ACA CCT GAA GCC CAA -3 |
| Efcab1 2for | 5- GGG AAA GTG AGG GCA AGC AAG AAA -3 |
| Efcab1 2rev | 5- CCA TCT GGC TCT GTA AGA TAA AGG G -3 |
| Efcab1 3for | 5- GGG AAA GTG AGG GCA AGC AAG AAA -3 |
| Efcab1 3rev | 5- CCA TCT GGC TCT GTA AGA TAA AGG G -3 |
| Ube2v2 for | 5- TGC CTT AGT CTC CCA ACC CAG TTT -3 |
| Ube2v2 rev | 5- TCA CCG CGA GCT CTG ACA -3 |
| Mcm4 for | 5- AGA CAA AGA CTT TAA GCG GTG GT -3 |
| Mcm4 rev | 5- GGC CTT TGG TGA TGT CCT AAC TCA -3 |
| Prkdc 1for | 5- AAG GTG GCA TTG CCC ACA GTT ATC -3 |
| Prkdc 1rev | 5- GTG CTA TTG TCC CGA GCA TGC AAA -3 |
| Prkdc 2for | 5- AAG GTG GCA TTG CCC ACA GTT ATC -3 |
| Prkdc 2rev | 5- GTG CTA TTG TCC CGA GCA TGC AAA -3 |
| Prkdc 3for | 5- GTG TCA CAG TGC ATG TAT GGA GGT -3 |
| Prkdc 3rev | 5- AGT GTG TCC AAG TCA CAG TGC CA -3 |
| Prkdc 4for | 5- TTC AAA CCA CCA CAG GAA CTA GGG -3 |
| Prkdc 4rev | 5- AGG GCT ATC ATG GGA GCC ATA CAT -3 |
| Prkdc 5for | 5- TAT TGC TCC AAA CCT CCC TCC AGT -3 |
| Prkdc 5rev | 5- GGT CCA TCT GCC TTA CTT GCA TGA -3 |
| Prkdc 6for | 5- AGA ATC CAG TGG TCC CTT AGC AGA -3 |
| Prkdc 6rev | 5- TGG CCT GTG GGT AGT TAT CAG CAA -3 |
| Cebpd for | 5- GCG AGG TGA CAG CCC AAC TT -3 |
| Cebpd rev | 5- AAA CTA GCG ATT CGG GCG GCG TCT -3 |
| 2310008H04Rik 1for | 5- TGT TGT ACA TGG GTG AGT GGC AGT -3 |
| 2310008H04Rik 1rev | 5- CCC AGT TTG TCT ATA CAG TGT CCT GC -3 |
| 2310008H04Rik 2for | 5- ATG GCT GGT AAG ATG GCT TCT CCA -3 |
| 2310008H04Rik 2rev | 5- ACA CAG GAT GAA AGG GAC CAC TCT -3 |

**Supplemental Methods Reference:**

1. Hallahan AR, Pritchard JI, Hansen S, Benson M, Stoeck *J, et a*l. (2004) The SmoA1 mouse model reveals that notch signaling is critical for the growth and survival of sonic hedgehog-induced medulloblastomas*. Cancer resear*ch 64(21):7794-7800.
